# Using host‐associated differentiation to track source population and dispersal distance among insect vectors of plant pathogens

**DOI:** 10.1111/eva.12733

**Published:** 2019-02-12

**Authors:** Gina Marie Angelella, Andy P. Michel, Ian Kaplan

**Affiliations:** ^1^ Department of Entomology Purdue University Lafayette Indiana; ^2^ Department of Entomology The Ohio State University Wooster Ohio; ^3^ Present address: Department of Horticulture Virginia Tech University, Eastern Shore Agricultural Research and Extension Center Painter Virginia

**Keywords:** *Aphis craccivora*, *Medicago sativa*, multilocus genotype, *Robinia pseudoacacia*, single nucleotide polymorphism, *Trifolium* spp.

## Abstract

Small, mobile insects are notoriously challenging to track across landscapes and manage in agricultural fields. However, genetic differentiation among insect populations and host plants acquired through host‐associated differentiation could be exploited to infer movement within crop systems and damage potential. Although many insects exhibit host‐associated differentiation, management strategies for insect vectors of plant pathogens assume a homogenous population. Nevertheless, phenotypic changes derived from host‐associated differentiation could manifest in altered behavior or physiology affecting the likelihood of vector–pathogen–plant interactions, or the subsequent efficiency of pathogen transmission. We used SNPs to assess genotypic structure and host‐associated differentiation in the cowpea aphid, *Aphis craccivora* Koch (Hemiptera: Aphididae). To do so, we sampled *A. craccivora* across the Midwestern United States. from two host plants, alfalfa (*Medicago sativa*) and black locust (*Robinia pseudoacacia*)—putative source populations for winged migrants. Simultaneously, we sampled winged *A. craccivora* landing in pumpkin fields where they transmit viruses. Structure analyses supported host‐associated differentiation by identifying two major genotypic groups: an alfalfa group containing a single multilocus genotype and a locust group containing all others. Winged locust‐group aphids landed at a much greater magnitude within focal fields during year 2 than year 1, while those in the alfalfa group remained fairly consistent. Spatial autocorrelation analyses indicated locust‐group aphid movement was characterized by small‐scale dispersal during year 2, likely originating from populations within 10 km. We also detected strong temporal differences in colonization from the two host plants. Early in the summer, most winged aphids (79.4%) derived from the locust group, whereas late in the summer more (58.3%) were from the alfalfa group. Because early crop growth stages are more susceptible to damage from aphid‐vectored viruses, these data implicate locust as the more important source and illustrate how host‐associated differentiation can be used to track dispersal and inform management of heterogeneous pest populations.

## 1 INTRODUCTION

Host‐associated differentiation (HAD) is a type of ecological isolation whereby herbivorous insects specialize on different plant species, creating genetic divergence among sympatric subpopulations (Berlocher & Feder, 2002; Scheffer & Hawthorne, 2007; Stireman, John, Nason, & Heard, 2005). This may eventually result in speciation (Abrahamson, Eubanks, Blair, & Whipple, 2001; Bush, 1969), but gene flow can prevent the complete isolation of subpopulations and thus HAD is a mechanism that generates and maintains intraspecific genetic diversity. Although HAD is a fundamental concept for understanding incipient species formation and basic evolutionary principles, it can also be applied as a tool to improve insect management in agricultural systems (Diehl & Bush, 1984; Medina, 2012). Numerous insect pests display HAD or related forms of population substructure (Antwi, Sword, & Medina, 2015; Bethenod et al., 2005; Brown, Frohlich, & Rosell, 1995; Pashley, 1986), yet their management strategies virtually always assume a homogenous population colonizing crops.

Accounting for predictable host races could prove especially useful for identifying the source populations and dispersal distances of small, mobile insects that are nearly impossible to otherwise track across agricultural landscapes. Aphids, for example, are minute even by insect standards and are known for their complex flight patterns, including the propensity to engage in long‐distance migrations (Loxdale et al., 1993). Importantly, aphids exhibit a suite of life history characteristics (e.g., asexual reproduction) that make them prone to exhibit HAD (Dickey & Medina, 2010), and many populations display some sort of underlying genetic structure according to host‐plant or geography (Delmotte, Leterme, Gauthier, Rispe, & Simon, 2002; Guillemaud, Mieuzet, & Simon, 2003; Loxdale & Lushai, 2007; Loxdale et al., 2011; Orantes, Wei Zhang, Mian, & Michel, 2012; Wilson, Sunnucks, Blackman, & Hales, 2002). However, few studies have used HAD‐mediated population differences to infer the source and movement of key plant pathogen vectors: one recent study used microsatellite loci to identify the originating host plants for pea aphids, *Acyrthosiphon pisum*, migrating into legume crops across the north‐western United States (Eigenbrode et al., 2016).

In this study, we used molecular markers to explore the roles of HAD and geographical scale across putative source populations in determining the spatial ecology of the cowpea aphid, *Aphis craccivora* Koch (Hemiptera: Aphididae), an asexually reproducing vector of plant viruses. Our prior research into the epidemiology of cucurbit crop viruses identified *A. craccivora* as the most important vector among a community of 53 aphid species alighting in fields (Angelella et al., 2015). While cucurbits are nonhosts for *A. craccivora* (i.e., they cannot survive and reproduce on these plants), winged alates can nevertheless introduce viruses through a stylet‐borne nonpersistent route of transmission. The source for these vectors is completely unknown, but *A*. *craccivora* is widely considered a specialist on legumes (Blackman, 2015), which likely cannot overwinter in the Midwest. Because this plant family (Fabaceae) is quite large, we focused primarily on two common legumes in the Midwestern United States that occur near agricultural areas and are frequently reported to contain *A*. *craccivora*: black locust (*Robinia pseudoacacia*) and alfalfa (*Medicago sativa*). Interestingly, prior research has shown that *A. craccivora* possess unique bacterial symbionts that maintain separate host‐associated populations for these two host plants (Brady & White, 2013; Wagner et al., 2015). We therefore expect that *A. craccivora* originating from locust are genetically distinct from those coming from alfalfa.

Determining the origin of winged *A. craccivora* that serve as crop virus vectors is potentially important for several reasons. One, legumes can serve as reservoirs for cucurbit viruses, including black locust (Laney, Avanzato, & Tzanetakis, 2012), alfalfa and clover (Brunt et al., 2017). Thus, aphids originating from these plants may acquire different viruses before alighting in fields. Two, vector competence varies with host plant associations in other aphid species (Coutts, Hawkes, & Jones, 2006; Gray, Smith, Barbierri, & Burd, 2002; McGrath & Harrison, 1995). The *A. craccivora* system is further complicated by the fact that migrants from locust and alfalfa possess different endosymbionts, which affect plant probing behaviors that mediate virus transmission (Angelella, Nalam, Nachappa, White, & Kaplan, 2017). Last, *A*. *craccivora* population dynamics and production of winged morphs likely vary between a crop, such as alfalfa, and an unmanaged tree like black locust*.* Alfalfa is regularly mown several times a year, which could allow populations to build up and induce dispersal during these disturbance events (Kennedy & Storer, 2000; Losey & Eubanks, 2000), generating pulses of migrating alates. In comparison, black locust seedlings—the preferred growth stage for aphids—are less disturbed and grow opportunistically in smaller, more widely distributed patches. Also, *A. craccivora* are commonly tended by mutualistic ants on locust, while this was almost never observed in alfalfa (G. Angelella, *personal observation*). This difference may be ecologically significant since ants usually suppress aphid wing formation (Müller, Williams, & Hardie, 2001).

Here, we describe the results from a 2‐year field analysis aimed at identifying the source of *A. craccivora* flying into focal pumpkin fields where they transmit economically important viruses. To do so, we sampled populations of *A. craccivora* on alfalfa, locust, and other fabaceous host plants (e.g., clover) at local and regional scales surrounding pumpkin fields. We then used single nucleotide polymorphisms (SNPs) to test for evidence of HAD and, simultaneously, compared the genetic structure of *A. craccivora* populations with migrant winged aphids flying into focal fields. Finally, we examined geographical patterns in genetic relatedness with spatial autocorrelation analyses to assess patterns in dispersal behavior, predicting that a tendency toward more frequent small‐scale movements among populations would lead to positive local, rather than regional, spatial autocorrelation.

## 2 METHODS

### 2.1 Aphid collection

**Figure 1:**
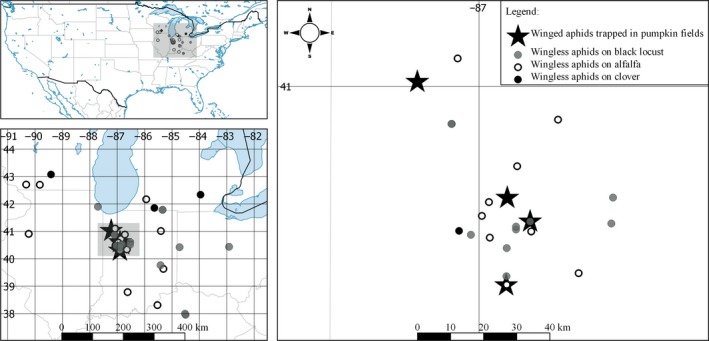
Map of *Aphis craccivora* collection sites (also see Supporting Information Table S1). Decimal degree latitudinal and longitudinal coordinates indicated

We trapped winged aphids landing in four focal pumpkin fields with pans (DiFonzo, Ragsdale, Radcliffe, Gudmestad, & Secor, 1997) in a 1:4 propylene glycol and water solution, collecting aphids once a week in 2012 (year 1) and 2013 (year 2). Twenty pan traps were set out in each field for 14 weeks after pumpkin plant emergence or transplant in fields and ending with the onset of plant senescence (6/19/2012–9/23/2012; 6/24/2013–9/23/2013). Focal fields were located in north‐west Indiana in Tippecanoe, White, and Jasper Cos. (Figure 1; Supporting Information Table S1). We identified *A*. *craccivora* from all alates trapped weekly within each field and stored them separately by field and collection date at −80°C in undiluted ethanol.

We collected apterae by hand from potential source populations on black locust and with sweep nets in alfalfa or clover (*Trifolium* spp. L.). Collections from potential source populations occurred within the duration of focal field sampling, except for clover which was only sampled in year 1 due to the difficulty in locating clover fields. Any temporal variation between alfalfa and locust collection dates largely results from variable periods of aphid colonization, as collection attempts were made throughout the sampling period until at least 20 individuals could be collected in a single sampling event. Source population locations were selected to represent a wide variety of distances both locally (ca. 3–50 km) and regionally (ca. 100–700 km) relative to the four focal pumpkin fields (Figure 1; Supporting Information Table S1). To include a more representative composition of aphid clones, or multilocus genotypes (MLGs), per population, we swept alfalfa or clover fields 10 times in 10 locations at least 30 m apart. Populations of *A*. *craccivora* on locust were found on seedlings, often concentrated on new growth on one location. Because populated seedlings were isolated, *A. craccivora* in locust populations were collected from a single plant at each location. All aphid samples were stored at −80°C in undiluted ethanol.

We extracted individual aphid DNA with DNeasy Blood and Tissue Kit (Qiagen, Hilden, Germany). Samples with DNA concentration <5 ng/μl were amplified with REPLI‐g Mini kit (Qiagen). In total, we extracted DNA from 503 aphids collected in year 1, and 465 aphids collected in year 2. We additionally extracted DNA following this protocol from 20 winged *A. craccivora* trapped in pan traps at two pumpkin field sites in 2010 and 2011 for RAD library preparation and sequencing. The pumpkin sites were located within the radius of the four focal fields sampled in years 1 and 2.

### 2.2 RAD library preparation, SNP identification and assessment of function

For RAD library preparation, we used 3 μg DNA from a pool of 20 winged *A. craccivora* trapped at two pumpkin field sites in 2010 and 2011. Library preparation followed protocol described by Etter and Johnson (2012) for RAD paired‐end sequencing. The DNA underwent a single digestion with the restriction enzyme EcoRI‐HF, after which P1 adaptors incorporating six barcodes, each 12 nucleotides in length, were ligated to digested DNA. Barcodes were selected to differ as much as possible. The ligated DNA fragments were then sheared to an average 500 bp in length, and size‐selected via gel electrophoresis to select samples 300–500‐bp in length. Next, PE‐P2 adaptors were ligated to DNA fragments. High‐fidelity PCR amplified the P1 and PE‐P2 adaptor‐ligated DNA fragments, after a test amplification to evaluate library quality. We then sequenced the library on an Illumina MiSeq platform, which produced 6,065,758 single reads passing the filter, with a read length of 250 bp. Raw reads were exported in fastq files. We conducted library preparation and sequencing at the OARDC Molecular‐Cellular Imaging Center.

The RAD tags were then used to identify de novo candidate SNPs with Stacks v. 1.19 (Catchen, Amores, Hohenlohe, Cresko, & Postlethwait, 2011; Catchen, Hohenlohe, Bassham, Amores, & Cresko, 2013), as we lacked a reference genome for *A*. *craccivora*. First, we demultiplexed and cleaned raw reads with the process_radtags program. We truncated reads to 224 bp, removed those with any uncalled bases, and discarded those with low‐quality scores. In all, we retained 5,347,646 high‐quality reads. Next, we ran the denovo_map.pl pipeline which utilizes ustacks to align matching sequences (i.e., putative alleles), cstacks to make a catalog of the consensus loci, and sstacks to match all of the samples in the population against the catalog. We allowed a maximum of two mismatches between stacks within individuals and a minimum depth of coverage of three nucleotides for ustacks, and allowed no mismatches between stacks between individuals for cstacks.

Fifty candidate SNPs were run across all *A*. *craccivora* DNA samples collected in years 1 and 2, of which seven candidate SNPs failed validation (LGC Genomics LLC, Beverly, MA). We subsequently identified both aphid samples and loci with >15% missing data using GenAlEx v6.5 program (Peakall & Smouse, 2006, 2012) and discarded them from the dataset. Loci with a minor allele frequency <0.05 in one or more populations were then identified using the poppr package (Kamvar, Brooks, & Grünwald, 2015; Kamvar, Tabima, & Grünwald, 2014) and R v3.2.2 statistical software program (R Core & Team, 2015) and discarded. Additionally, we identified four nonneutral loci undergoing balancing selection, and one locus (Ac44139) undergoing positive selection using the LOSITAN program (Beaumont & Nicols, 1996; Supporting Information Figure S1). Upon examination, there was markedly greater homozygosity at locus Ac44139 in locust‐origin populations. The locus Ac44139 sequence was similar to an *Ac*. *pisum* transposon (based on BlastX), but was not found in an *A*. *craccivora* transcriptome (J. White, *unpublished data*). Regardless, we conducted analyses using neutral loci only as those undergoing selection can disrupt gene frequency calculations such as Wright’s *F*_ST_ (Beaumont & Nicols, 1996). Ultimately, the dataset included 817 aphid individuals and 21 loci.

Lastly, we explored the possibility of SNP effects on virus epidemiology by checking for alignment between SNP sequences and an *A*. *craccivora* transcriptome (J. White, *unpublished data*), and running a BLASTX search on those highly similar sequences (alignment identification >98%) to identify putative functions. We then compared open reading frames within highly similar transcriptome sequences with each SNP variant with ORF‐Finder (NCBI) to predict whether they would change resultant protein products.

### 2.3 Genetic diversity and polymorphism

We used the Paetkau assignment test (Paetkau, Slade, Burden, & Estoup, 2004) in GenAlEx to assign samples to groups. GenAlEx also matched individual aphid samples to MLGs; however, missing data can inflate the number of MLGs by reducing matches. To correct for this, we also manually assigned samples to MLG by “best estimate,” assigning an MLG only after eliminating all other possible matches (Gitzendanner, Weekley, Germain‐Aubrey, Soltis, & Soltis, 2012). We calculated pairwise codominant genetic distance between MLGs to assess the total number of differing alleles across loci. To assess genetic diversity, we followed recommendations made by Arnaud‐Haond, Duarte, Alberto, and Serrão (2007) in selecting diversity parameters appropriate for clonal organisms: we used the poppr package in R statistical software program to calculate Simpson complement (D^*^), Simpson evenness indices (*V*), and the slope of the Pareto distribution (*c*), and calculated genotypic richness (*R*:* R *= (*G *− 1)/(*N *− 1), where *G* = number of MLGs and *N* = population size). Again, following recommendations for analysis of clonal organisms by Arnaud‐Haond et al. (2007), we recalculated all of the statistical analyses after removing repeat MLGs within a population to exclude the effect of clonality unless otherwise stated. Observed (*H*_o_) and expected heterozygosity (*H*_e_), inbreeding coefficient (*F*_is_), allele frequencies, and deviation from Hardy–Weinberg equilibrium were calculated with GenAlEx. To assess the likelihood of clonality or recombination within populations sampled, indicating sexual or asexual reproduction within populations, we calculated, a ${\overset{¯}{r}}_{d}$ modified version of the index of association (*I*_A_) robust to data with multiple loci (Agapow & Burt, 2001), using the poppr package.

### 2.4 Population genetic structure

To visualize genetic structure among and within aphid populations, we used STRUCTURE v2.3.4, which infers populations among samples and can identify migrant or admixed samples (Falush, Stephens, & Pritchard, 2003; Pritchard, Stephens, & Donnelly, 2000). Because STRUCTURE methods assume HWE, only the datasets with all repeat MLGs removed within populations underwent the analysis. We used a burn‐in length of 50,000 and 100,000 Monte Carlo Markov‐Chain repetitions to test *K* = 2–21 number of genetic groups, and each value of *K* was replicated 10 times. We ran admixture models with LOCPRIOR, which uses sample location data to inform the assignment of clusters in studies with fewer markers, individuals or weaker structure without increasing spurious structure detection (Hubisz, Flush, Stephens, & Pritchard, 2009; Pritchard, Stephens, & Donnelly, 2010). The probable value of *K* was generated using the Evanno, Regnaut, and Goudet (2005) method with STRUCTURE HARVESTER program (Earl, 2012). The graphical output was visualized with Distruct v1.1 (Rosenberg, 2004). We calculated the proportion of individuals within each population identifying within each STRUCTURE‐assigned group. To quantify genetic differentiation between STRUCTURE‐assigned groups, we calculated Wright’s *F*_ST_ (Wright, 1978).

### 2.5 Spatial/Temporal patterns in migrant aphids

We assessed the relationship of codominant genotypic distance and geographical distance among sample sites with global spatial autocorrelation in GenAlEx, generating 999 bootstrapped coefficient values (*r*) with 999 random permutations. We included geographical distance class end points of 0, 10, 20, 50, 100, 200, 300, 400, and >400 km in analyses. The 0 km distance class was included to distinguish between aphids collected from the same population (0 km geographical distance separation) vs. from nearby populations separated by some distance within 10 km. Because it is thought most aphid dispersal occurs over short distances (Loxdale et al., 1993), incremental distance units were much closer together to identify the most influential scale within 50 km. Beyond the local scale, we increased distance classes by 100 km, ensuring multiple paired comparisons were present in each distance class. Because weather patterns and other related external factors can influence aphid population dynamics, and these patterns varied between years, years 1 and 2 were analyzed separately. Aphids were analyzed by STRUCTURE‐assigned group.

Although varying sample collection dates can lead to variations in self‐population assignment, with clonal diversity within populations either increasing (Michel, Zhang, Jung, Kang, & Rouf Mian, 2009; Orantes et al., 2012) or decreasing (Komazaki et al., 2011; Sunnucks, Barro, Lushai, Maclean, & Hales, 1997) over time, levels of self‐population assignment in this study were very low across all populations (Supporting Information Table S2). As such, we chose not to assess frequency of self‐assignment as a function of collection date. However, we assessed the temporal relationship of host‐associated MLGs in focal field alatae (migrants) with a logistical regression of migrant alfalfa‐ or locust‐group identity assigned by STRUCTURE analysis, by a full factorial cross of year and early or late seasonal occurrence. The “early” bin contained samples collected in weeks 1–7 of the sampling period, and the “late” bin, weeks 8–14. All migrants were included in the analysis.

## 3 RESULTS

### 3.1 SNP transcription

Fourteen out of the 21 SNP loci were highly similar to sequences in an *A*. *craccivora* transcriptome search (Supporting Information Table S3A). Four of these transcriptome sequences were highly similar in a BlastX search with *Ac. pisum*,* Diuraphis noxia*, and *Myzus persicae* sequences predicting proteins as well (Supporting Information Table S3B). One locus, Ac107071, was highly similar to a sequence within the *A*. *craccivora* transcriptome and also with a predicted filaggrin or filaggrin‐like protein in *Ac*. *pisum*,* D*. *noxia*, and *M. persicae*. An additional locus, Ac88299, was highly similar to an *A. craccivora* transcriptome sequence, which in turn matched uncharacterized proteins in *Ac. pisum*,* D. noxia*, and *M*. *persicae*. The SNP variants induced open reading frame changes altering an unnamed protein product within the transcriptome sequence matching locus Ac88299, but not at any other loci.

### 3.2 Polymorphism and genetic diversity

Before the removal of repeat MLGs, all loci in 11 populations were not in HWE, including all alfalfa‐origin populations and one locust‐origin population (Supporting Information Table S4). The remaining black locust populations had almost entirely monomorphic loci. After repeat MLGs (assigned by best estimate) were removed, there was only a single locus in a focal field population, “*K*,” not in equilibrium or monomorphic (Supporting Information Table S5). Seven repeat MLGs were identified, with memberships of 428, 256, 73, 18, 6, 3, and 3 individuals (Table 1). The remaining 31 MLGs occurred in only one individual across all populations.

**Table 1:** Aphid multilocus genotype (MLG) assignment using best estimate method, by year and population. Letter categories indicate names of an MLG to which multiple aphids were assigned, while aphids with a unique MLG are indicated by the “Single MLG” category

Population	W	R	Q	P	O	L	D	Single MLG
Year 1 (2012)								
Migrant	0	0	0	0	0	13	0	4
Alfalfa	0	0	0	0	0	162	0	0
Locust	161	0	0	0	7	1	16	0
Clover	20	1	0	2	0	25	0	1
Clover + Alfalfa	0	0	0	0	0	22	0	0
Year 2 (2013)								
Migrant	4	4	0	0	6	8	1	18
Alfalfa	0	0	3	0	1	182	1	3
Locust	71	0	0	0	59	15	0	5

Among the nonmigratory aphids, the dominant MLG was found largely in alfalfa‐origin populations: 84.5% of the populations within which the largest MLG was identified were of alfalfa origin, and 98.6% of alfalfa‐origin aphids had this MLG. The second most dominant MLG was found primarily in locust‐origin populations: 90.6% of the populations within which this MLG was identified were of locust origin, and 70.3% of locust‐origin aphids had this MLG. Of the remaining repeat MLGs, two were largely locust‐origin (20.0% of locust‐origin aphids), one of alfalfa origin (0.9% of alfalfa‐origin aphids), and two were found only in clover aphid populations. Three repeat MLGs (W, O, D) found in locust were only slightly distinct, with genetic distances of 1–6, and 28 of 31 single MLGs had total allele differences of <10 (Table 2). The dominant locust MLG (W) and dominant alfalfa MLG (L) had the greatest genetic distance between them (Table 2).

**Table 2:** Pairwise genetic distance (total number of differing alleles across loci) between each best estimate MLG and repeat MLGs. Single MLGs found in only one individual are numbered 1–31, while repeat MLGs found in at least two individuals are indicated by a letter

	L	W	D	O	P	Q	R
L:	0						
W:	21	0					
D:	16	5	0				
O:	20	1	6	0			
P:	18	11	14	10	0		
Q:	17	16	19	15	9	0	
R:	15	14	15	13	11	10	0
1:	21	22	25	23	13	8	12
2:	20	13	16	14	4	9	13
3:	18	13	16	12	12	17	7
4:	20	15	18	14	6	9	15
5:	18	15	20	14	8	1	9
6:	15	10	13	9	5	4	6
7:	17	8	11	7	1	10	8
8:	19	22	23	21	11	6	8
9:	7	12	15	11	9	4	6
10:	14	11	14	10	4	7	5
11:	17	16	17	17	5	6	8
12:	20	15	16	16	12	11	9
13:	19	20	21	17	9	14	10
14:	16	13	14	12	4	9	5
15:	22	21	22	18	6	13	13
16:	22	11	12	10	14	19	11
17:	17	4	7	3	7	10	10
18:	16	17	18	16	4	3	9
19:	20	11	16	12	12	7	9
20:	18	11	14	10	2	7	13
21:	20	19	22	20	12	7	13
22:	18	19	22	18	6	3	7
23:	21	14	19	13	7	8	16
24:	18	15	18	16	10	3	9
25:	17	12	15	13	5	8	8
26:	17	8	13	7	3	6	6
27:	15	14	15	13	7	6	12
28:	20	1	6	2	12	17	15
29:	15	10	13	11	7	8	4
30:	27	16	21	17	15	20	24
31:	16	13	16	12	4	3	9

The dominant alfalfa‐origin MLG exhibited excess heterozygosity, whereas a heterozygote deficit was found in the dominant locust‐origin MLG, which had many monomorphic loci. The dominant alfalfa‐origin MLG differentiated from all other MLGs in heterozygosity at nine loci. Locus Ac88299 was heterozygous in all aphids but the dominant alfalfa‐origin MLG contained one SNP while all others expressed the alternative nucleotide. Overall, levels of genotypic diversity were low, with focal field migrant populations, a clover field (“BuckCreek”) and a black locust population (“OH”) exhibiting the highest relative diversity (Supporting Information Table S2). All multilocus disequilibrium estimates (${\overset{¯}{r}}_{d}$) were significant (*p* < 0.01), indicating sexual recombination seems to be absent within *A. craccivora* across all populations (Supporting Information Table S6).

### 3.3 Population genetic structure

STRUCTURE analysis results suggested a probability value of *K* = 2 distinct genetic groups (Supporting Information Figure S2). There were no differences in the optimal number of population clusters or in individuals’ group assignment comparing datasets with repeat MLGs removed after assignment by conservative vs. best estimate approach. There was very little evidence of admixture in the manually assigned dataset, but several individuals in the conservative dataset showed admixed lineages. However, due to inclusion of “unique” MLGs containing missing loci in the conservative dataset, the admixture could be a byproduct of genotyping error generated associated with missing data (Reeves, Bowker, Fettig, Tembrock, & Richards, 2016).

**Figure 2:**
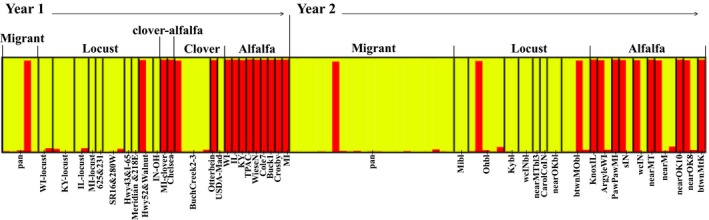
STRUCTURE diagram of *K* = 2 genetic groups, assigned to individual *Aphis craccivora.* Sampled populations are delineated and labeled below, with individual MLGs within each population represented by vertical bars of equal size, colored to indicate STRUCTURE‐assigned group membership (red* *= alfalfa group, yellow* *= locust group). Actual population sample type and collection year are indicated above the graph. Analysis was run under an admixture model, with combined year 1 (2012) and year 2 (2013) datasets, excluding repeat MLGs after best estimate MLG assignment

**Figure 3:**
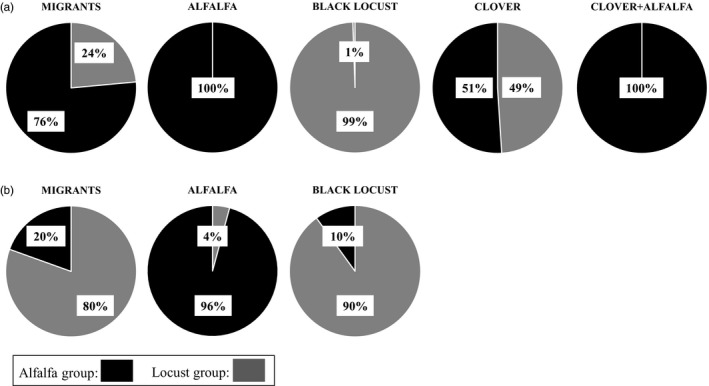
Proportion of STRUCTURE‐assigned group among individual wingless *Aphis craccivora* collected by host plant (“alfalfa,” “black locust,” “clover,” “clover + alfalfa” mix) and winged *Aphis craccivora* trapped within focal pumpkin fields (“migrants”). (a) Year 1 (2012) dataset, (b) Year 2 (2013) dataset

The STRUCTURE diagram predicts population MLG assortment into two groups, largely separated between alfalfa‐ and locust‐origin populations (Figure 2): membership of one group contained the MLG “L,” and membership of the second group contained all other MLGs. As “L” was the dominant MLG of alfalfa‐origin aphids in both years, the first STRUCTURE‐assigned group will be referred to as the alfalfa group, and the second as the locust group. A separation by group between locust‐ and alfalfa‐origin populations is clear in year 1 and somewhat mixed in year 2. The STRUCTURE diagram reflects the MLGs assigned to each group, however; examining the proportion of individuals of each group within populations illustrates the strength of separation by group between alfalfa and black locust populations in year 2 as well (Figure 3). Clover populations represented intermediate group membership, while clover + alfalfa mix populations were entirely derived from the alfalfa group (Figures 2 and 3). Migrant populations of *A. craccivora* landing in pumpkin fields derived from both groups (Figures 2 and 3). The alfalfa and locust groups had an *F*_ST_ of 0.156, indicating great genetic differentiation (Wright, 1978).

### 3.4 Spatial/Temporal patterns in migrant aphids

**Figure 4:**
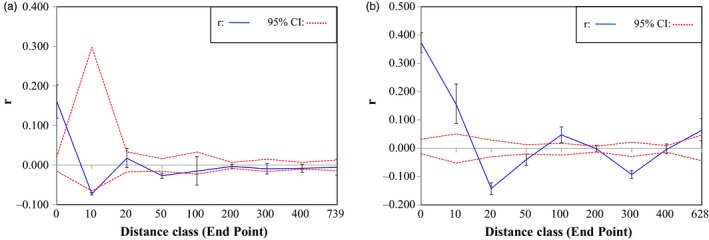
Spatial autocorrelation analyses of *Aphis craccivora* genetic relatedness by geographical distance for locust‐group aphids, as assigned by STRUCTURE analysis. Error bars indicate *r* estimates from 999 bootstraps. Red dashed lines indicate ±95% confidence intervals. (a) Year 1 (2012) dataset, (b) Year 2 (2013) dataset

Only aphids assigned by STRUCTURE analysis to the locust group were analyzed, as all members of the alfalfa group were genetically identical and therefore lacked spatial genetic structure. The degree of spatial patterning within locust‐group aphids varied by year. In year 1, aphids exhibited positive spatial autocorrelation within a given population at 0 km (*r* = 0.16, *p* = 0.001), and very weak negative effects across populations at 10, 50, and 400 km as well (*r *= −0.073, *p = *0.001; *r *= −0.027, *p* = 0.003; *r *= −0.008, *p* = 0.045, respectively; Figure 4a). In year 2, aphids exhibited positive spatial autocorrelation again within a given population at 0 km (*r* = 0.37, *p* = 0.001), but also across populations at 10 km, and 100 km (*r* = 0.15, *p* = 0.001; *r* = 0.048, *p* = 0.001, respectively), and exhibited negative spatial autocorrelation at 20, 50, and 300 km (*r *= −0.14, *p* = 0.001; *r *= −0.040, *p* = 0.002; *r *= −0.093, *p* = 0.001, respectively; Figure 4b).

Results were similar following analyses of locust‐group datasets excluding repeat MLGs. Aphids were marginally significant within a given population at 0 km in both years (year 1: *r* = 0.21, *p* = 0.053; year 2: *r* = 0.087, *p* = 0.053; Supporting Information Figure S3A,B). In year 2, aphids also showed positive spatial autocorrelation across populations within 10 km (*r* = 0.19, *p* = 0.037), and in year 1 populations within 300 and >400 km (*r* = 0.25, *p* = 0.023; *r* = 0.23, *p* = 0.026, respectively). Negative spatial autocorrelation occurred in year 1 populations within 400 km (*r *= −0.084, *p* = 0.038) and in year 2 populations within 20 km and 300 km (*r *= −0.126, *p* = 0.007; *r *= −0.10, *p* = 0.019, respectively).

The membership of migrant aphids landing within focal fields to either the locust‐group or alfalfa group varied significantly between years and between early and late seasonal timing (year: *χ* ^2^ **= 4.16, *SE* = 0.97, *p* = 0.041; seasonal timing: *χ* ^2^ **= 3.90, *SE* = 0.84, *p* = 0.048). In total, there were 13 alfalfa group *A. craccivora* alates in year 1 migrant populations and 8 in year 2, with relatively more landing in focal fields late‐season (year 1:69.2%; year 2:62.5%; Table 3). Locust‐group migrants varied between years with a total of four individuals trapped in focal fields in year 1, evenly distributed between early‐ and late‐season (50.0%). In year 2, 33 total alates landed in focal fields, with a greater percentage arriving early in the season (75.8%; Table 2).

**Table 3:** Total number of winged *Aphis craccivora* trapped in lower north‐western Indiana focal pumpkin fields, and number of MLGs grouped with predominately black locust (*N*_LG_) or alfalfa (*N*_AG_) populations early and late in the growing season (early: weeks 1–7; late: weeks 8–14): MLGs assigned by STRUCTURE analysis

	Early			Late		
	*N*	*N*_LG_	*N*_AG_	*N*	*N*_LG_	*N*_AG_
Year 1 (2012)	6	2	4	11	2	9
Year 2 (2013)	28	25	3	13	8	5

## 4 DISCUSSION

Genetic grouping of *A. craccivora* individuals clearly varied by alfalfa or locust host association according to STRUCTURE analysis, making it possible to identify putative host plant sources of winged aphid migrants landing in focal fields. The host plant origin of migrants was influenced by both year and early/late seasonal timing. Alfalfa‐group migrants tended to occur later in the season with relatively less year‐to‐year variation in magnitude (relative ratio of early‐to‐late‐season migrants was ca. 1:2; *n*_year1_ **= 13, *n*_year2_ **= 8), whereas locust‐derived aphids differed in early:late proportion and magnitude by year (1:1 early‐to‐late ratio in year 1, *n*_year1_ **= 4; 25:8 in year 2, *n*_year2_ **= 33). Thus, alfalfa may be a relatively minor contributor of migrants in focal pumpkin fields with low total numbers overall and biased toward late‐season activity, and the contribution of migrants from black locust may fluctuate more by year, but years with greater migrant production could generate more early‐season dispersal.

The pattern has important implications for virus epidemiology. Black locust, alfalfa, and clover are host to several pumpkin viruses. Locust is host to papaya ringspot virus type W (PRSV‐W) and watermelon mosaic virus type 2 (WMV; Laney et al., 2012), and alfalfa and at least two species of clover (*T*. *incarnatum, T*. *pratense*) are susceptible to WMV (Brunt et al., 2017). Cucumber mosaic virus was also isolated from alfalfa and white clover (*T. repens*) in Wisconsin (Mueller, Groves, & Gratton, 2012) and is an additional aphid‐vectored virus periodically found in pumpkins in the midwestern region of the United States (Jossey & Babadoost, 2008; Walters, Kindhart, Hobbs, & Eastburn, 2003). Crops are more susceptible to reductions in yield due to virus infection in earlier stages of growth; thus, vectors interacting with crops earlier in the growing season are of far greater concern.

Although the relatively low number of migrating aphids we trapped in focal fields is not unrealistic, it does raise some concern regarding sample size. The total *A. craccivora* trapped per field was comparable to that of a previous study in area pumpkin fields (Angelella et al., 2015), and to that of a similar study of *Ac. pisum* migrant population genetics in peas (Eigenbrode et al., 2016). However, because migrant trap catches can be quite low within crop fields, it is possible that patterns in MLG composition could be impacted by sample size and the ability to obtain a representative sample. As such, we would recommend investigating whether increasing the number and/or density of pan traps per crop field could lead to a more optimal sampling effort for migrant vectors such as *A. craccivora*. If, however, aphids are preferentially landing in pan traps relative to the surrounding crop canopy (Boiteau, 1990), it is possible that roughly the same total abundance could be trapped per field irrespective of trap density. In fact, we obtained roughly similar trap catches to that of Angelella et al. (2015) and Eigenbrode et al. (2016) despite using a greater number of pan traps per field: 20 field^−1^ compared to 5 field^−1^ and 3 field^−1^, respectively. Notwithstanding, care should be taken to optimize the migrant vector sampling method.

Our results support patterns of genetic relatedness and differentiation among locust‐group *A. craccivora* across populations by geographical distance and year. Alfalfa‐group *A. craccivora* were genetically identical both within and among collection sites, and thus exhibited no genotypic spatial structure. Results of year 2 locust‐origin aphids may indicate a connection between small‐scale geographical movement and migrant alightment in focal fields. Positive genetic structure within 10 km and differentiation at 20 km suggest small‐scale movement among populations was common in year 2, corresponding with a large number of locust‐origin migrants in focal fields. In contrast, the differentiation of year 1 locust‐origin aphids within even the small geographical scale of 10 km may suggest a lack of small‐scale dispersal, which corresponds with infrequent migrant arrival in focal fields. This smaller‐scale dispersal range could make repositioning crop fields to alleviate influx of migrant aphids more feasible, potentially helping growers to move away from areas prone to repeated virus infection. Locust‐group aphids also showed some indication of moderate migratory dispersal capabilities, as evidenced by differentiation at moderate geographical distances of 300–400 km in both years. This would be consistent with a spring recolonization of the Midwest from southern overwintering populations requiring regional‐scale movement. However, these overwintering populations would not likely be located at the relatively high latitudes within the 300–400 km range of the focal pumpkin fields (Dykstra et al., 2014).

*Aphis craccivora* populations on alfalfa were dominated by a single MLG characterized by excess heterozygosity. While locust populations had one dominant MLG, an additional MLG was moderately represented among individuals in year 2, and several other MLGs were also present at low frequency in both year 1 and year 2. In contrast to the dominant alfalfa‐origin MLG, the dominant locust‐origin MLG exhibited homozygote excess. There are multiple possibilities for these outcomes, two of which include inbreeding (Simon & Hebert, 1995), or null alleles (Foltz, 1986). These possibilities are unlikely for the *A. craccivora* in this study because we confirmed the likelihood of obligate parthenogenesis and strictly asexual reproduction, and we selectively removed all loci suspected to contain null alleles. Another potential explanation is the Wahlund effect, which can occur when subpopulation structure leads to artificially decreased heterozygosity (Wahlund, 1928); for example, if subpopulations are separated geographically or temporally, genetic drift could subsequently occur within these subpopulations decreasing heterozygosity (Prugnolle & De Meeûs, 2010). The Wahlund effect is possible: spatial autocorrelation analysis indicated geographical isolation among locust‐group populations around 20 and 300 km apart in year 2. Remaining possibilities include side‐effects of life cycle, and clonal selection. There is some suggestion that asexual anholocyclic lifecycle might favor the production of homozygote excess (Simon et al., 1999), because patterns of mutation might accumulate and persist throughout the overwintering and recolonization of a single plant‐host (Loxdale et al., 2011). Conversely, others have speculated that asexual populations tend to favor heterozygosity by accumulating mutations (Balloux, Lehmann, & Meeȗs, 2003; Delmotte et al., 2002; Harrison John & Mondor, 2011; Lynch, 1984; Vorburger, Lancaster, & Sunnucks, 2003) while sexual populations select for homozygosity in favored alleles (Hales, Tomuik, Wöhrmann, & Sunnucks, 1997; Simon & Hebert, 1995; Simon et al., 1999).

Nevertheless, the formation of predominant “superclones” can indicate relative fitness advantage (Vorburger et al., 2003). The concept of clonal amplification (Sunnucks et al., 1997) predicts clones with greater fitness will reproduce more rapidly to eventually outnumber other clones. This concept has found support in several aphid species, including *Myzus persicae* (Vorburger 2005), *Aphis glycines* (Wenger, Monica Ramstad, Mian, & Michel, 2014), *Aphis nerii* (Harrison John & Mondor, 2011), and *Ac. pisum* (Eigenbrode et al., 2016). Interestingly, authors of one study of aphids on tansy (*Tanacetum vulgare* L.) found no evidence of dominant clones and contrasted this to *M. persicae* and other pest species of intensively cultivated monocultures (Loxdale et al., 2011). If managed monoculture crops exert a stronger selective pressure on aphid MLGs, it would follow that aphid specialists in alfalfa would experience stronger selective pressure than those on unmanaged black locust seedlings. Unmanaged locusts also likely present a more varied nutritional profile and host quality; thus, naturally occurring black locust diversity may be relatively high (Chang, Bongarten, & Hamrick, 1998), and additionally, black locusts are thought to associate with a high diversity of rhizobia symbionts leading to variations in N fixation, total N, and plant growth (Batzli, Graves, & Berkum, 1992). The frequency of unique MLGs within focal field migrants also suggests that *A. craccivora* may be arriving from other populations, or even host plants other than alfalfa or black locust. However, it is also likely that mitotic mutations during clonal reproduction have resulted in multiple MLGs descending from the same clonal lineage if they exhibit low genetic distance (differences in alleles length; Arnaud‐Haond et al., 2007). Indeed, at least two repeat MLGs found in locust populations and 28/31 single MLGs were only slightly different from more numerically dominant MLG, suggesting they may belong to the same multilocus lineage (MLL). Thus, it is likely that the vast majority of migrants derived from an alfalfa‐associated or black locust‐associated genotype.

Genetic grouping by locust‐ or alfalfa‐association is compelling in light of recent evidence of distinct locust‐associated and alfalfa‐host‐associated populations driven by facultative symbiont infection (Wagner et al., 2015). *Aphis craccivora* populations on black locust are usually infected with the facultative endosymbiont, *Arsenophonus*, while those on alfalfa and other plant hosts are not (Brady & White, 2013). Wagner et al. (2015) found that *Arsenophonus* enhances aphid performance on black locust while decreasing it on other host plants, essentially facilitating its use as a host plant. They also observed *A. craccivora* clonal lines originating on alfalfa were especially well‐adapted to alfalfa: while locust‐origin clonal lines infected with *Arsenophonus* significantly outperformed on locust compared to alfalfa, alfalfa‐origin clonal lines transfected with *Arsenophonus* performed equally well on locust and alfalfa. It would be interesting to assess facultative endosymbiont infection relative to population genotypic structure and dispersal among host‐plant populations. The endosymbionts are horizontally transferred (Oliver, Degnan, Burke, & Moran, 2010; Sandström, Russell, White, & Moran, 2001), which could facilitate a sustained relationship between clonal populations and black locust, resulting in the accumulation of adaptive mutations in an MLG. In fact, recent research showed that genetic lineage, rather than endosymbiont association or host‐plant diet, drives the toxicity of black locust‐origin *A*. *craccivora* to the predator *Harmonia axyridis*, while alfalfa‐origin lineages are nontoxic and vulnerable to predation (White, McCord, Jackson, Dehnel, & Lenhart, 2017). The alfalfa “superclone,” in contrast, could not be attributed to facultative endosymbiont infection.

It is possible that host race formation within locust‐ and alfalfa‐associated populations has also generated phenotypic changes to behavior or physiology impacting virus transmission efficiency, and/or the likelihood of interacting with virus reservoirs and susceptible crop plants. Although it was evident that many of the study SNP sequences are being transcribed in *A*. *craccivora*, it is unclear at present whether they would result in phenotypic differentiation. But this would not be unprecedented. For example, regional populations of *Schizaphis graminum* (Rondani) within the United States varied in their host‐plant associations and ability to transmit five viruses that cause barley yellow dwarf disease (Gray et al., 2002). The authors found that the poorest virus vectors were cyclically parthenogenetic, holocyclic populations, and speculated that because one might expect higher rates of genetic diversity in those populations due to sexual recombination and random mating, it could follow that vector competence is more readily retained in obligately parthenogenetic, anholocyclic *S. graminum* if it is a recessive trait. The obligately parthenogenetic nature of *A. craccivora* reproduction could similarly facilitate the retention of MLGs with enhanced vector competence. Follow‐up studies could examine how relative transmission efficiencies vary among *A. craccivora* alfalfa‐ and locust‐associated clonal types.

## 5 CONCLUSION

We observed a pattern of distinct genotypic *A. craccivora* grouping by alfalfa‐ or black locust‐origin, suggesting HAD has generated two host races. Aphids placed into the predominantly alfalfa group by STRUCTURE analysis possessed a single MLG, which was by far the dominant MLG found in aphid populations on alfalfa. Aphids placed in the locust group largely possessed one dominant MLG, but were more diverse and contained several additional MLGs. Due to the lack of diversity among alfalfa aphids, we could not detect genetic structure relative to geographical distance; however, locust aphids supported a prediction for small‐scale dispersal in year 2, with some evidence of moderate migratory dispersal capabilities in both years. Due to evidence for an appreciable dispersal capability, the potential for generating large numbers of migrants, and the early‐season timing of dispersal flights, our data suggest that locust‐origin migrants are more important vectors in the pumpkin virus pathosystem.

## CONFLICT OF INTEREST

None declared.

## DATA ARCHIVING STATEMENT

All raw RAD library sequences are available at NCBI SRA: BioProject accession #PRJNA503177, BioSample accession #SAMN10354042. The validated SNPs developed in this study as well as the genotypic, geographical, population and host‐plant or pan collection source data have been deposited in Dryad at https://doi.org/10.5061/dryad.3560pr3.
