# Supplementary figures and images for: Using host‐associated differentiation to track source population and dispersal distance among insect vectors of plant pathogens
Source: Evol Appl. 2019 Feb 12;12(4):692–704. doi: 10.1111/eva.12733 (PMC6439873; doi:10.1111/eva.12733)

# Fst/He

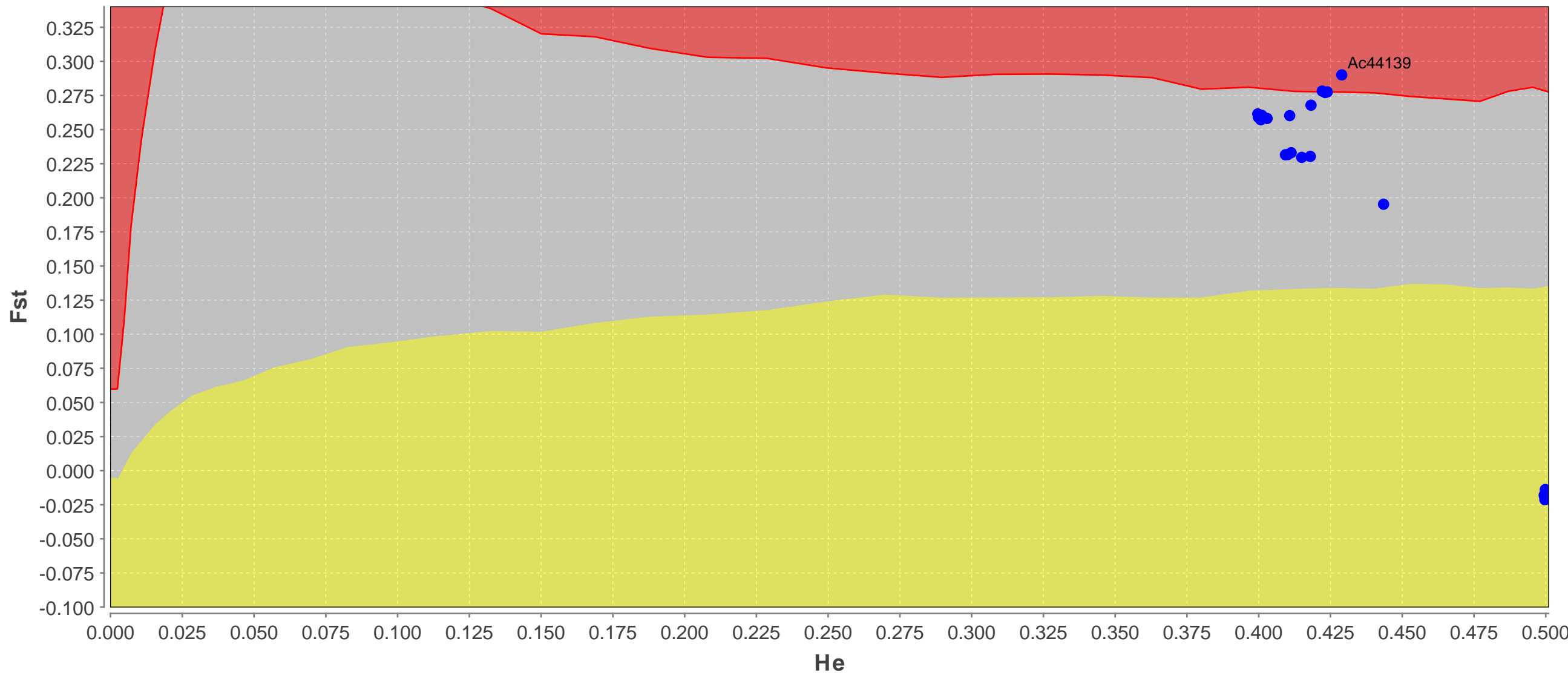

Supplement: Supplementary file 1 [file EVA-12-692-s001.pdf]

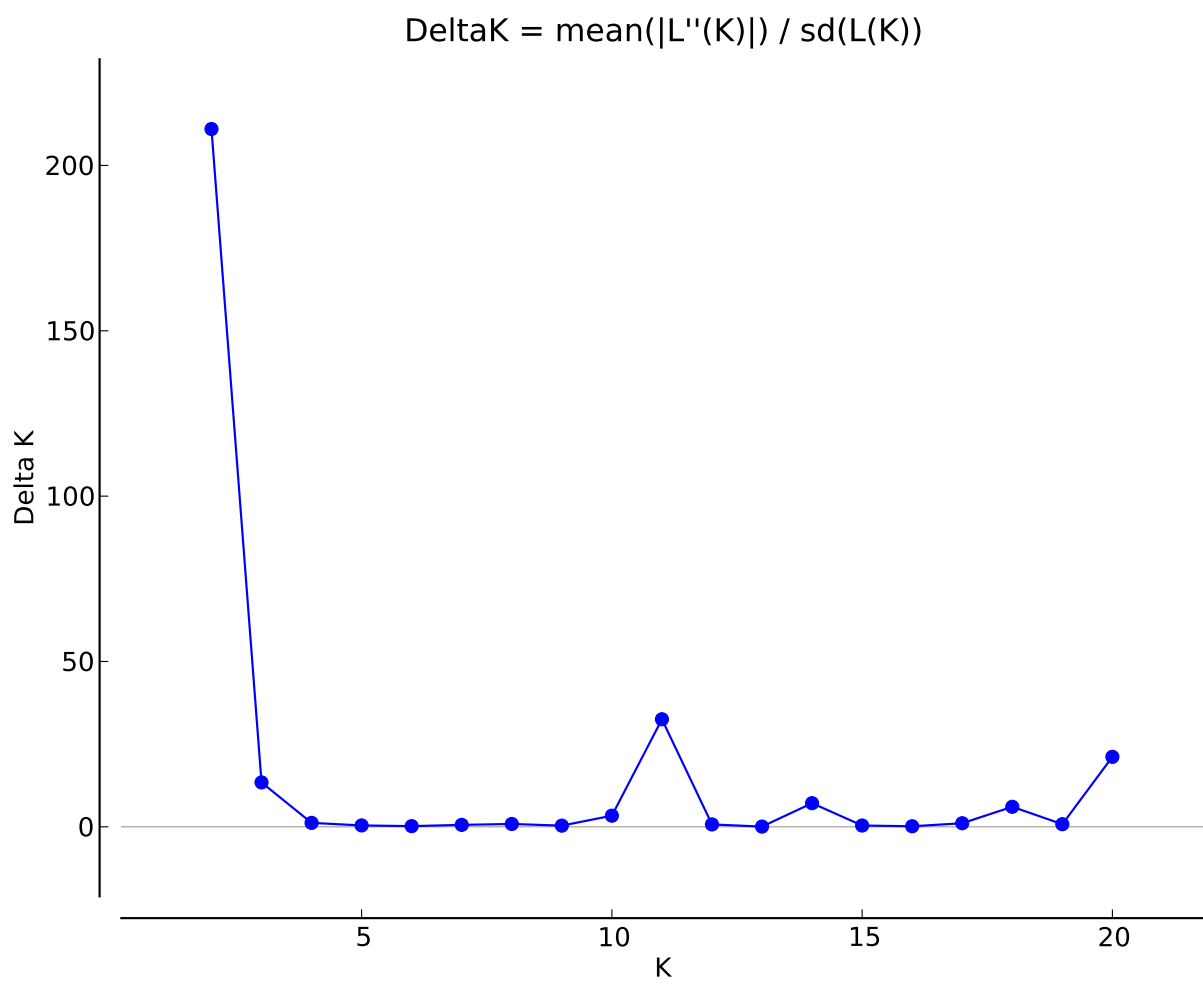

Supplement: Supplementary file 2 [file EVA-12-692-s002.pdf]

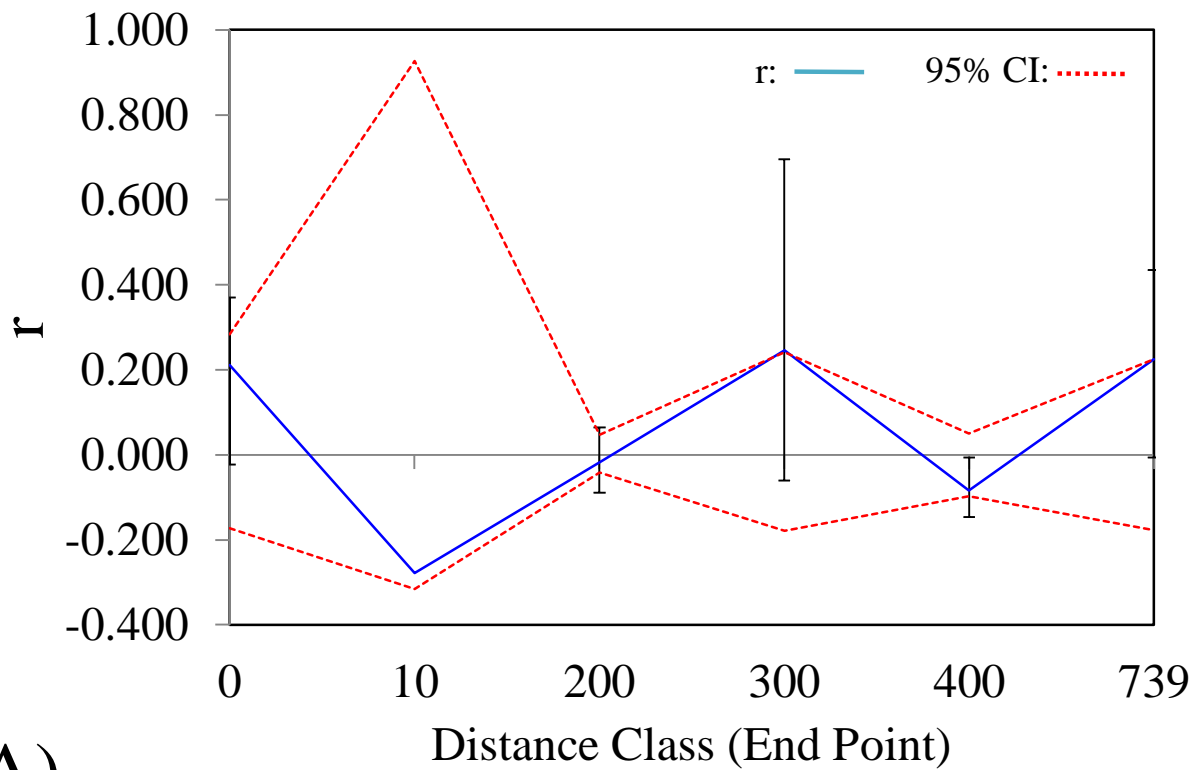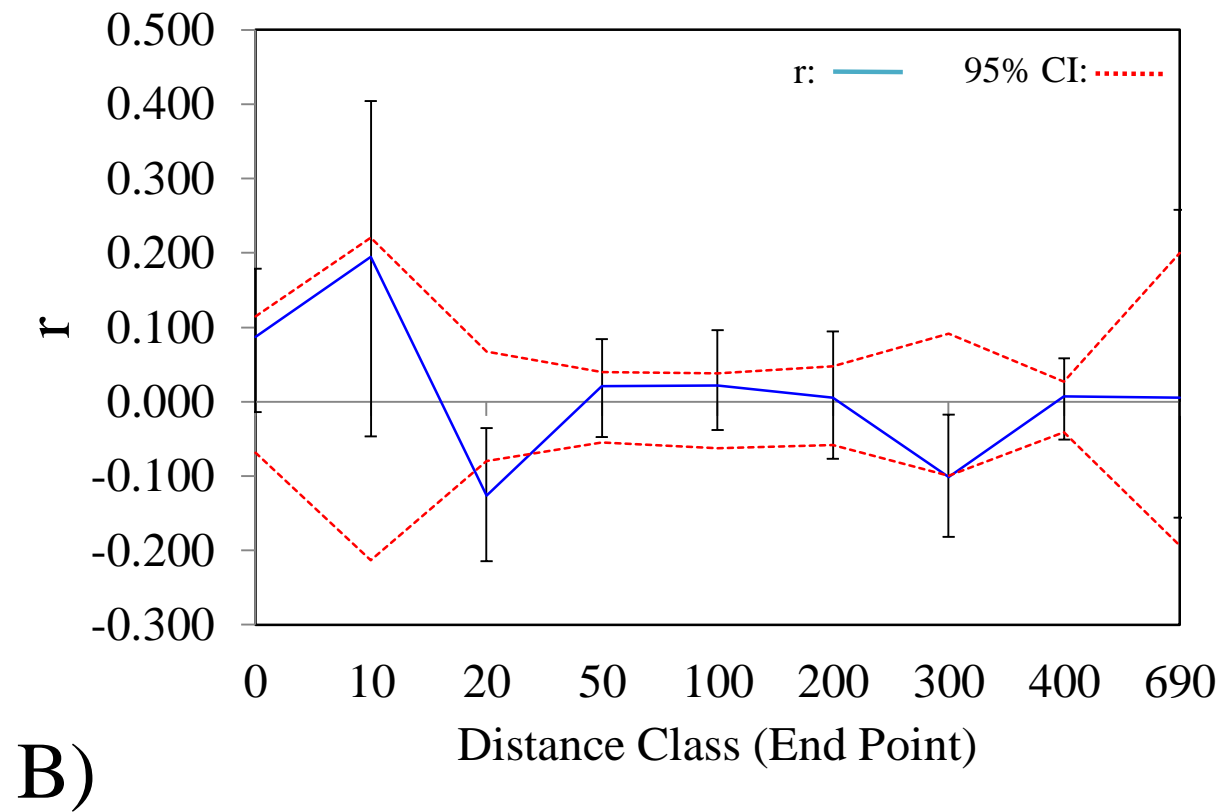

Supplement: Supplementary file 3 [file EVA-12-692-s003.pdf]
