# Supplementary material for: Using host‐associated differentiation to track source population and dispersal distance among insect vectors of plant pathogens
Source: Evol Appl. 2019 Feb 12;12(4):692–704. doi: 10.1111/eva.12733 (PMC6439873; doi:10.1111/eva.12733)
Supplement: Supplementary file 4 [file EVA-12-692-s004.docx]

**Table S1.** Collection location, date, population type, and sample size of *Aphis craccivora* used in year 1 and year 2 analyses.

|  | Pop. | Type | Date | | N | Lat. | Long. |
| --- | --- | --- | --- | --- | --- | --- | --- |
| Year 1 | Pan: K | migrant | 19-Jun—3-Sept-2012 | | 4 | 40.605 | -86.900 |
|  | Pan: M | migrant | 19-Jun—3-Sept-2012 | | 7 | 40.295 | -86.904 |
|  | Pan: Mt | migrant | 19-Jun—3-Sept-2012 | | 4 | 41.016 | -87.219 |
|  | Pan: O | migrant | 19-Jun—3-Sept-2012 | | 2 | 40.521 | -86.819 |
|  | WI-locust | black locust | | 31-Aug-2012 | 16 | 40.521 | -86.819 |
|  | KY-locust | black locust | | 21-Jun-2012 | 19 | 37.955 | -84.503 |
|  | IL-locust | black locust | | 23-Jun-2012 | 20 | 41.899 | -87.705 |
|  | MI-locust | black locust | | 16-Aug-2012 | 20 | 41.784 | -85.344 |
|  | 625&231 | black locust | | 23-Jul-2012 | 20 | 40.327 | -86.903 |
|  | 16&280W | black locust | | 23-Jul-2012 | 19 | 40.867 | -87.097 |
|  | Hw43&I-65 | black locust | | 6-Aug-2012 | 14 | 40.493 | -86.869 |
|  | Mer&218E | black locust | | 25-Jul-2012 | 20 | 40.606 | -86.525 |
|  | Hw52&Wal | black locust | | 5-Jul-2012 | 17 | 40.474 | -87.029 |
|  | IN-OH | black locust | | 22-Aug-2012 | 20 | 40.421 | -84.723 |
|  | MI-clover | clover-alfalfa | | 16-Aug-2012 | 12 | 41.853 | -85.644 |
|  | Chelsea | clover-alfalfa | | 17-Aug-2012 | 10 | 42.337 | -83.957 |
|  | BuckCreek | clover | 7-Aug-2012 | | 15 | 40.485 | -86.815 |
|  | Otterbein | clover | 6-Aug-2012 | | 15 | 40.488 | -87.071 |
|  | USDA-Mad | clover | 31-Aug-2012 | | 19 | 43.075 | -89.418 |
|  | WI | alfalfa | 31-Aug-2012 | | 19 | 42.706 | -90.324 |
|  | IL | alfalfa | 11-Jul-2012 | | 19 | 40.908 | -90.232 |
|  | KY | alfalfa | 11-Jul-2012 | | 10 | 38.307 | -85.531 |
|  | TPAC | alfalfa | 6-Aug-2012 | | 20 | 40.297 | -86.902 |
|  | WieseN | alfalfa | 20-Jul-2012 | | 18 | 40.590 | -86.965 |
|  | Cole7 | alfalfa | 25-Jul-2012 | | 20 | 40.464 | -86.962 |
|  | Buck1 | alfalfa | 7-Aug-2012 | | 20 | 40.486 | -86.815 |
|  | Crosby | alfalfa | 9-Jul-2012 | | 17 | 40.882 | -86.720 |
|  | MI | alfalfa | 16-Aug-2012 | | 19 | 41.011 | -85.405 |
| Year 2 | Pan: K | migrant | 24-Jun—23-Sept-2013 | | 11 | 40.605 | -86.900 |
|  | Pan: M | migrant | 24-Jun—23-Sept-2013 | | 14 | 40.295 | -86.904 |
|  | Pan: Mt | migrant | 24-Jun—23-Sept-2013 | | 9 | 41.016 | -87.219 |
|  | Pan: O | migrant | 24-Jun—23-Sept-2013 | | 7 | 40.521 | -86.819 |
|  | IL | alfalfa | 13-Aug-2013 | | 20 | 40.908 | -90.232 |
|  | WI | alfalfa | 12-Aug-2013 | | 20 | 42.699 | -89.827 |
|  | MI | alfalfa | 25-Aug-2013 | | 19 | 42.169 | -85.941 |
|  | sIN | alfalfa | 11-Sep-2013 | | 15 | 38.782 | -86.621 |
|  | wcIN | alfalfa | 11-Sep-2013 | | 20 | 39.629 | -85.315 |
|  | nearMT | alfalfa | 20-Aug-2013 | | 20 | 41.099 | -87.076 |
|  | nearM | alfalfa | 16-Jul-2013 | | 20 | 40.338 | -86.647 |
|  | nearOK10 | alfalfa | 4-Sep-2013 | | 19 | 40.541 | -86.990 |
|  | nearOK8 | alfalfa | 20-Aug-2013 | | 18 | 40.590 | -86.965 |
|  | btwnMtK | alfalfa | 16-Sep-2013 | | 19 | 40.717 | -86.865 |
|  | MIbl | black locust | 23-Jun-2013 | | 18 | 41.784 | -85.344 |
|  | OH | black locust | 9-Sep-2013 | | 18 | 40.436 | -82.916 |
|  | KY | black locust | 28-Sep-2013 | | 20 | 37.994 | -84.526 |
|  | wcINbl | black locust | 11-Sep-2013 | | 18 | 39.764 | -85.418 |
|  | nearMTbl3 | black locust | 12-Jul-2013 | | 20 | 40.867 | -87.098 |
|  | CarolCo | black locust | 6-Sep-2013 | | 20 | 40.514 | -86.531 |
|  | nearOK | black locust | 15-Jul-2013 | | 20 | 40.503 | -86.869 |
|  | btwnMO | black locust | 4-Sep-2013 | | 17 | 40.427 | -86.902 |
