# Supplementary material for: Using host‐associated differentiation to track source population and dispersal distance among insect vectors of plant pathogens
Source: Evol Appl. 2019 Feb 12;12(4):692–704. doi: 10.1111/eva.12733 (PMC6439873; doi:10.1111/eva.12733)
Supplement: Supplementary file 5 [file EVA-12-692-s005.docx]

**Table S2.** Individual *Aphis craccivora* assignments to self (self P) or other populations (other P), number of matching, unique, and total MLGs within each population, and population genetic diversity parameters genotypic richness (*R*), Simpson complement (*D^1^*), Simpson evenness (*V*), and the slope of the Pareto distribution (*c*).

|  |  | | ASSIGNMENT | | | | GENOTYPES | | | | | |  | | |  |  | | |  | |  |
| --- | --- | --- | --- | --- | --- | --- | --- | --- | --- | --- | --- | --- | --- | --- | --- | --- | --- | --- | --- | --- | --- | --- |
|  | Pop. | self | | other | | self P/N | # match | | # unique | | total | | R | | V | | | | | | C | |
| Year 1 | Pan | | 0 | | 17 | 0 | 1_a_ | 2_b_ | 4 _a_ | 6 _b_ | 5 _a_ | 8 _b_ | 0.25 _a_ | 0.44_b_ | -0.13_a_ | | | -1.15 _b_ | 0.90 | | | |
| (2012) | WI-locust | | 5 | | 11 | 0.31 | 2 _a_ | 2 _b_ | 0 _a_ | 0 _b_ | 2 _a_ | 2 _b_ | 0.07 _a_ | 0.07 _b_ | 1.09 _a_ | | | 1.09 _b_ | 0.88 | | | |
|  | KY-locust | | 0 | | 19 | 0 | 3 _a_ | 4 _b_ | 0 _a_ | 0 _b_ | 3 _a_ | 4 _b_ | 0.11 _a_ | 0.17 _b_ | 0.34 _a_ | | | 0.15 _b_ | 0.59 | | | |
|  | IL-locust | | 0 | | 20 | 0 | 2 _a_ | 2 _b_ | 0 _a_ | 2 _b_ | 2 _a_ | 4 _b_ | 0.05 _a_ | 0.16 _b_ | 1.48 _a_ | | | 0.88 _b_ | 0.49 | | | |
|  | MI-locust | | 20 | | 0 | 1.00 | 1 _a_ | 3 _b_ | 0 _a_ | 0 _b_ | 1 _a_ | 3 _b_ | 0 _a_ | 0.11 _b_ | *—* ^ϯ^ _a_ | | | 1.22 _b_ | 0.38 | | | |
|  | 625&231 | | 0 | | 20 | 0 | 1 _a_ | 2 _b_ | 0 _a_ | 0 _b_ | 1 _a_ | 2 _b_ | 0 _a_ | 0.05 _b_ | *—* ^ϯ^ _a_ | | | 1.89 _b_ | 0.24 | | | |
|  | 16&280W | | 0 | | 19 | 0 | 3 _a_ | 4 _b_ | 0 _a_ | 0 _b_ | 3 _a_ | 4 _b_ | 0.11 _a_ | 0.17 _b_ | 0.27 _a_ | | | 0.09 _b_ | 0.57 | | | |
|  | Hw43&I-65 | | 0 | | 14 | 0 | 1 _a_ | 1 _b_ | 0 _a_ | 0 _b_ | 1 _a_ | 1 _b_ | 0 _a_ | 0.00 _b_ | *—* ^ϯ^ _a_ | | | *—* ^ϯ^ _b_ | N/A | | | |
|  | Mer&218E | | 0 | | 20 | 0 | 1 _a_ | 1 _b_ | 0 _a_ | 0 _b_ | 1 _a_ | 1 _b_ | 0 _a_ | 0.00 _b_ | *—* ^ϯ^ _a_ | | | *—* ^ϯ^ _b_ | N/A | | | |
|  | Hw52&Wal | | 0 | | 17 | 0 | 2 _a_ | 3 _b_ | 0 _a_ | 2 _b_ | 2 _a_ | 5 _b_ | 0.06 _a_ | 0.25 _b_ | 1.16 _a_ | | | 0.41 _b_ | 0.63 | | | |
|  | IN-OH | | 0 | | 20 | 0 | 1 _a_ | 3 _b_ | 0 _a_ | 0 _b_ | 1 _a_ | 3 _b_ | 0 _a_ | 0.11 _b_ | *—* ^ϯ^ _a_ | | | 1.22 _b_ | 0.38 | | | |
|  | MI-clover | | 12 | | 0 | 1.00 | 1 _a_ | 2 _b_ | 0 _a_ | 0 _b_ | 1 _a_ | 2 _b_ | 0 _a_ | 0.09 _b_ | *—* ^ϯ^ _a_ | | | 1.80 _b_ | 0.29 | | | |
|  | Chelsea | | 0 | | 10 | 0 | 1 _a_ | 2 _b_ | 0 _a_ | 0 _b_ | 1 _a_ | 2 _b_ | 0 _a_ | 0.11 _b_ | *—* ^ϯ^ _a_ | | | 1.74 _b_ | 0.32 | | | |
|  | BuckCreek | | 0 | | 15 | 0 | 4 _a_ | 4 _b_ | 1 _a_ | 2 _b_ | 5 _a_ | 6 _b_ | 0.29 _a_ | 0.36 _b_ | -0.21 _a_ | | | -0.55 _b_ | 0.76 | | | |
|  | Otterbein | | 0 | | 15 | 0 | 1 _a_ | 1 _b_ | 0 _a_ | 0 _b_ | 1 _a_ | 1 _b_ | 0 _a_ | 0.00 _b_ | *—* ^ϯ^ _a_ | | | *—* ^ϯ^ _b_ | N/A | | | |
|  | USDA-Mad | | 0 | | 19 | 0 | 1 _a_ | 1 _b_ | 0 _a_ | 0 _b_ | 1 _a_ | 1 _b_ | 0 _a_ | 0.00 _b_ | *—* ^ϯ^ _a_ | | | *—* ^ϯ^ _b_ | N/A | | | |
|  | WI | | 0 | | 19 | 0 | 1 _a_ | 1 _b_ | 0 _a_ | 0 _b_ | 1 _a_ | 1 _b_ | 0 _a_ | 0.00 _b_ | *—* ^ϯ^ _a_ | | | *—* ^ϯ^ _b_ | N/A | | | |
|  | IL | | 0 | | 19 | 0 | 1 _a_ | 3 _b_ | 0 _a_ | 1 _b_ | 1 _a_ | 4 _b_ | 0 _a_ | 0.17 _b_ | *—* ^ϯ^ _a_ | | | 0.85 _b_ | 5.00 | | | |
|  | KY | | 0 | | 10 | 0 | 1 _a_ | 3 _b_ | 0 _a_ | 0 _b_ | 1 _a_ | 3 _b_ | 0 _a_ | 0.22 _b_ | *—* ^ϯ^ _a_ | | | 0.78 _b_ | 0.53 | | | |
|  | TPAC | | 0 | | 20 | 0 | 1 _a_ | 3 _b_ | 0 _a_ | 0 _b_ | 1 _a_ | 3 _b_ | 0 _a_ | 0.11 _b_ | *—^a^* _a_ | | | 1.07 _b_ | 0.37 | | | |
|  | WieseN | | 0 | | 18 | 0 | 1 _a_ | 1 _b_ | 0 _a_ | 0 _b_ | 1 _a_ | 1 _b_ | 0 _a_ | 0.00 _b_ | *—* ^ϯ^ _a_ | | | *—* ^ϯ^ _b_ | N/A | | | |
|  | Cole7 | | 0 | | 20 | 0 | 1 _a_ | 1 _b_ | 0 _a_ | 0 _b_ | 1 _a_ | 1 _b_ | 0 _a_ | 0.00 _b_ | *—* ^ϯ^ _a_ | | | *—* ^ϯ^ _b_ | N/A | | | |
|  | Buck1 | | 0 | | 20 | 0 | 1 _a_ | 3 _b_ | 0 _a_ | 1 _b_ | 1 _a_ | 4 _b_ | 0 _a_ | 0.16 _b_ | *—* ^ϯ^ _a_ | | | 0.88 _b_ | 0.49 | | | |
|  | Crosby | | 0 | | 17 | 0 | 1 _a_ | 2 _b_ | 0 _a_ | 0 _b_ | 1 _a_ | 2 _b_ | 0 _a_ | 0.06 _b_ | *—* ^ϯ^ _a_ | | | 1.63 _b_ | 0.34 | | | |
|  | MI | | 0 | | 19 | 0 | 1 _a_ | 2 _b_ | 0 _a_ | 1 _b_ | 1 _a_ | 3 _b_ | 0 _a_ | 0.11 _b_ | *—* ^ϯ^ _a_ | | | 1.21 _b_ | 0.39 | | | |
| Year 2 | Pan | | 21 | | 20 | 0.51 | 5 _a_ | 5 _b_ | 18 _a_ | 20 _b_ | 23 _a_ | 25 _b_ | 0.55 _a_ | 0.60 _b_ | -3.81 _a_ | | | -5.09 _b_ | 1.47 | | | |
| (2013) | IL | | 0 | | 20 | 0 | 1 _a_ | 2 _b_ | 0 _a_ | 3 _b_ | 1 _a_ | 5 _b_ | 0 _a_ | 0.21 _b_ | *—* ^ϯ^ _a_ | | | 0.45 _b_ | 0.54 | | | |
|  | WI | | 0 | | 20 | 0 | 1 _a_ | 2 _b_ | 1 _a_ | 1 _b_ | 2 _a_ | 3 _b_ | 0.05 _a_ | 0.11 _b_ | 1.68 _a_ | | | 1.22 _b_ | 0.38 | | | |
|  | MI | | 0 | | 19 | 0 | 1 _a_ | 3 _b_ | 0 _a_ | 0 _b_ | 1 _a_ | 3 _b_ | 0 _a_ | 0.11 _b_ | *—* ^ϯ^ _a_ | | | 1.21 _b_ | 0.39 | | | |
|  | sIN | | 0 | | 15 | 0 | 1 _a_ | 2 _b_ | 1 _a_ | 2 _b_ | 2 _a_ | 4 _b_ | 0.07 _a_ | 0.21 _b_ | 1.29 _a_ | | | 0.65 _b_ | 0.56 | | | |
|  | wcIN | | 0 | | 20 | 0 | 2 _a_ | 6 _b_ | 0 _a_ | 1 _b_ | 2 _a_ | 7 _b_ | 0.05 _a_ | 0.32 _b_ | 0.95 _a_ | | | -0.04 _b_ | 0.74 | | | |
|  | nearMT | | 0 | | 20 | 0 | 1 _a_ | 1 _b_ | 0 _a_ | 1 _b_ | 1 _a_ | 2 _b_ | 0 _a_ | 0.05 _b_ | *—* ^ϯ^ _a_ | | | 1.89 _b_ | 0.24 | | | |
|  | nearM | | 0 | | 20 | 0 | 2 _a_ | 1 _b_ | 1 _a_ | 4 _b_ | 3 _a_ | 5 _b_ | 0.11 _a_ | 0.21 _b_ | 0.90 _a_ | | | 0.59 _b_ | 0.58 | | | |
|  | nearOK10 | | 0 | | 19 | 0 | 1 _a_ | 2 _b_ | 0 _a_ | 0 _b_ | 1 _a_ | 2 _b_ | 0 _a_ | 0.06 _b_ | *—* ^ϯ^ _a_ | | | -0.24 _b_ | 0.24 | | | |
|  | nearOK8 | | 0 | | 18 | 0 | 2 _a_ | 4 _b_ | 0 _a_ | 0 _b_ | 2 _a_ | 4 _b_ | 0.06 _a_ | 0.18 _b_ | 0.90 _a_ | | | 0.36 _b_ | 0.57 | | | |
|  | btwnMtK | | 0 | | 19 | 0 | 1 _a_ | 1 _b_ | 0 _a_ | 1 _b_ | 1 _a_ | 2 _b_ | 0 _a_ | 0.06 _b_ | *—* ^ϯ^ _a_ | | | 1.88 _b_ | 0.24 | | | |
|  | MIbl | | 0 | | 18 | 0 | 2 _a_ | 2 _b_ | 0 _a_ | 1 _b_ | 2 _a_ | 3 _b_ | 0.06 _a_ | 0.12 _b_ | 1.43 _a_ | | | 1.01 _b_ | 0.39 | | | |
|  | OH | | 0 | | 18 | 0 | 1 _a_ | 1 _b_ | 4 _a_ | 7 _b_ | 5 _a_ | 8 _b_ | 0.24 _a_ | 0.41 _b_ | -0.02 _a_ | | | -0.86 _b_ | 0.87 | | | |
|  | KY | | 18 | | 2 | 0.90 | 2 _a_ | 3 _b_ | 0 _a_ | 3 _b_ | 2 _a_ | 6 _b_ | 0.05 _a_ | 0.26 _b_ | 1.11 _a_ | | | 0.30 _b_ | 0.66 | | | |
|  | wcINbl | | 0 | | 18 | 0 | 2 _a_ | 4 _b_ | 0 _a_ | 2 _b_ | 2 _a_ | 6 _b_ | 0.06 _a_ | 0.29 _b_ | 0.86 _a_ | | | -0.05 _b_ | 0.65 | | | |
|  | nearMTbl3 | | 0 | | 20 | 0 | 1 _a_ | 2 _b_ | 0 _a_ | 0 _b_ | 1 _a_ | 2 _b_ | 0 _a_ | 0.05 _b_ | *—* ^ϯ^ _a_ | | | 1.89 _b_ | 0.24 | | | |
|  | CarolCo | | 0 | | 20 | 0 | 1 _a_ | 3 _b_ | 0 _a_ | 1 _b_ | 1 _a_ | 4 _b_ | 0 _a_ | 0.16 _b_ | *—* ^ϯ^ _a_ | | | 0.88 _b_ | 0.49 | | | |
|  | nearOK | | 0 | | 20 | 0 | 2 _a_ | 2 _b_ | 0 _a_ | 1 _b_ | 2 _a_ | 3 _b_ | 0.05 _a_ | 0.11 _b_ | 1.33 _a_ | | | 0.93 _b_ | 0.40 | | | |
|  | btwnMO | | 0 | | 17 | 0 | 3 _a_ | 3 _b_ | 1 _a_ | 3 _b_ | 4 _a_ | 6 _b_ | 0.19 _a_ | 0.31 _b_ | -0.05 _a_ | | | -0.56 _b_ | 0.79 | | | |

_a_=calculated with manual multilocus lineage assignment

_b_=calculated with conservative multilocus lineage assignment

^ϯ^=not applicable due to monomorphic population (total # MLGs=1)
