# Supplementary material for: Using host‐associated differentiation to track source population and dispersal distance among insect vectors of plant pathogens
Source: Evol Appl. 2019 Feb 12;12(4):692–704. doi: 10.1111/eva.12733 (PMC6439873; doi:10.1111/eva.12733)
Supplement: Supplementary file 6 [file EVA-12-692-s006.docx]

**Table S3.** Results of search through *Aphis craccivora* transcriptome for alignments with SNP sequences: A) Alignments within *Aphis craccivora* transcriptome, B) *Aphis craccivora* transcriptome sequences highly aligned with SNP sequence (alignment identification >98%) with all available sequences within the BlastX search engine, and predicted functions of highly scored (≥500) matches.

| **SNP ID** | ***Aphis craccivora* Transcriptome Sequence^*^** | **%ID** | **Alignment Length** | **Mismatches** | **E value** | **Bitscore** |
| --- | --- | --- | --- | --- | --- | --- |
| Ac25986 | TRINITY_DN94319_c0_g1_i1 | 99.55 | 224 | 1 | 1.00E-113 | 411 |
| Ac25986 | TRINITY_DN81104_c0_g1_i1 | 99.55 | 224 | 1 | 1.00E-113 | 411 |
| Ac106241 | TRINITY_DN62397_c5_g2_i2 | 99.55 | 224 | 1 | 1.00E-113 | 411 |
| Ac65895 | TRINITY_DN60920_c1_g2_i1 | 99.55 | 224 | 1 | 1.00E-113 | 411 |
| Ac82746 | TRINITY_DN64031_c1_g1_i1 | 99.11 | 225 | 1 | 5.00E-112 | 405 |
| Ac91605 | TRINITY_DN27910_c0_g1_i1 | 99.43 | 175 | 1 | 2.00E-86 | 320 |
| Ac102292 | TRINITY_DN65458_c9_g1_i1 | 97.37 | 228 | 2 | 2.00E-106 | 387 |
| Ac45540 | TRINITY_DN60034_c2_g1_i1 | 99.55 | 224 | 1 | 1.00E-113 | 411 |
| Ac105372 | TRINITY_DN60992_c2_g1_i1 | 99.55 | 224 | 1 | 1.00E-113 | 411 |
| Ac24056 | TRINITY_DN63260_c8_g1_i1 | 99.55 | 224 | 1 | 1.00E-113 | 411 |
| Ac19923 | TRINITY_DN58758_c2_g2_i1 | 99.55 | 224 | 1 | 1.00E-113 | 411 |
| Ac99882 | TRINITY_DN61304_c3_g1_i1 | 99.55 | 224 | 1 | 1.00E-113 | 411 |
| Ac88299 | TRINITY_DN65462_c16_g1_i2 | 99.36 | 157 | 1 | 2.00E-76 | 287 |
| Ac94989 | TRINITY_DN57921_c11_g1_i1 | 97.8 | 227 | 2 | 2.00E-107 | 390 |
| Ac107071 | TRINITY_DN105352_c0_g1_i1 | 99.55 | 224 | 1 | 1.00E-113 | 411 |
| Ac107071 | TRINITY_DN51319_c0_g1_i1 | 100 | 144 | 0 | 3.00E-70 | 267 |

**^*^*Aphis craccivora* transcriptome data provided by J. White**

| ***Aphis craccivora* transcriptome sequence^*^** | **Aligned sequence** | **% ID** | **% Positives** | **E value** | **Bitscore** | **Function** |
| --- | --- | --- | --- | --- | --- | --- |
| TRINITY_DN51319_c0_g1_i1 | XP_015377594.1 | 85.34 | 90.78 | 0 | 563 | PREDICTED: filaggrin-like [*Diuraphis noxia*] |
|  | XP_003243748.1 | 85.14 | 90.33 | 0 | 544 | PREDICTED: filaggrin [*Acyrthosiphon pisum*] |
|  | XP_022170793.1 | 85.78 | 90.04 | 0 | 563 | Filaggrin-like [*Myzus persicae*] |
| TRINITY_DN65462_c16_g1_i2 | XP_015372660.1 | 88.96 | 91.94 | 8.25e-168 | 537 | PREDICTED: uncharacterized protein LOC107167920 [*Diuraphis noxia*] |
|  | XP_001952607.2 | 89.85 | 93.13 | 5.93e-163 | 524 | PREDICTED: uncharacterized protein LOC100162487 [*Acyrthosiphon pisum*] |
|  | XP_022171920.1 | 88.96 | 91.64 | 2.2e-160 | 518 | Uncharacterized protein LOC11034836 [*Myzus persicae*] |

**^*^*Aphis craccivora* transcriptome data provided by J. White**
