# Supplementary material for: Using host‐associated differentiation to track source population and dispersal distance among insect vectors of plant pathogens
Source: Evol Appl. 2019 Feb 12;12(4):692–704. doi: 10.1111/eva.12733 (PMC6439873; doi:10.1111/eva.12733)
Supplement: Supplementary file 7 [file EVA-12-692-s007.docx]

**Table S4.** Polymorphism and heterozygosity of year 1 (2012) and year 2 (2013) *Aphis craccivora* populations, as assessed by Hardy Weinberg Equilibrium: full dataset.

| **Population** | Column1 | **Ac101026** | **Ac102292** | **Ac105372** | **Ac106241** | **Ac107071** | **Ac19923** | **Ac24056** | **Ac24766** | **Ac25986** | **Ac33020** | **Ac45540** | **Ac65895** | **Ac66996** | **Ac80798** | **Ac82746** | **Ac85822** | **Ac88299** | **Ac91605** | **Ac9471** | **Ac94989** | **Ac99882** |
| --- | --- | --- | --- | --- | --- | --- | --- | --- | --- | --- | --- | --- | --- | --- | --- | --- | --- | --- | --- | --- | --- | --- |
| **pan_2012** | *H_o_* | 0.765 | 0.765 | 0.765 | 0.867 | 0.765 | 0.765 | 0.882 | 0.733 | 0.765 | 0.813 | 0.824 | 0.941 | 0.800 | 0.765 | 0.824 | 0.750 | 0.824 | 0.882 | 0.765 | 0.750 | 0.765 |
|  | *H_e_* | 0.472 | 0.472 | 0.472 | 0.491 | 0.472 | 0.472 | 0.493 | 0.491 | 0.472 | 0.482 | 0.484 | 0.498 | 0.480 | 0.472 | 0.498 | 0.469 | 0.484 | 0.493 | 0.493 | 0.469 | 0.472 |
|  | *F_is_* | **-0.619** | **-0.619** | **-0.619** | **-0.765** | **-0.619** | **-0.619** | **-0.789** | -0.493 | **-0.619** | **-0.684** | **-0.700** | **-0.889** | **-0.667** | **-0.619** | **-0.653** | **-0.600** | **-0.700** | **-0.789** | **-0.551** | **-0.600** | **-0.619** |
| **WI-locust_2012** | *H_o_* | 0.313 | 0.313 | 0.000 | 0.000 | 0.000 | 0.000 | 0.313 | 0.000 | 0.000 | 0.000 | 0.000 | 0.000 | 0.000 | 0.000 | 0.000 | 0.000 | 0.000 | 0.313 | 0.000 | 0.313 | 0.000 |
|  | *H_e_* | 0.264 | 0.264 | 0.000 | 0.000 | 0.000 | 0.000 | 0.264 | 0.000 | 0.000 | 0.000 | 0.000 | 0.000 | 0.000 | 0.000 | 0.000 | 0.000 | 0.000 | 0.264 | 0.000 | 0.264 | 0.000 |
|  | *F_is_* | -0.185 | -0.185 | #N/A | #N/A | #N/A | #N/A | -0.185 | #N/A | #N/A | #N/A | #N/A | #N/A | #N/A | #N/A | #N/A | #N/A | #N/A | -0.185 | #N/A | -0.185 | #N/A |
| **KY-locust_2012** | *H_o_* | 0.263 | 0.263 | 0.000 | 0.158 | 0.000 | 0.000 | 0.263 | 0.000 | 0.000 | 0.000 | 0.000 | 0.000 | 0.000 | 0.000 | 0.000 | 0.000 | 0.000 | 0.263 | 0.000 | 0.263 | 0.000 |
|  | *H_e_* | 0.229 | 0.229 | 0.000 | 0.145 | 0.000 | 0.000 | 0.229 | 0.000 | 0.000 | 0.000 | 0.000 | 0.000 | 0.000 | 0.000 | 0.000 | 0.000 | 0.000 | 0.229 | 0.000 | 0.229 | 0.000 |
|  | *F_is_* | **-0.152** | **-0.152** | **#N/A** | **-0.086** | **#N/A** | **#N/A** | **-0.152** | **#N/A** | **#N/A** | **#N/A** | **#N/A** | **#N/A** | **#N/A** | **#N/A** | **#N/A** | **#N/A** | **#N/A** | **-0.152** | **#N/A** | **-0.152** | **#N/A** |
| **IL-locust_2012** | *H_o_* | 0.050 | 0.050 | 0.000 | 0.000 | 0.000 | 0.000 | 0.050 | 0.000 | 0.000 | 0.000 | 0.000 | 0.000 | 0.000 | 0.000 | 0.000 | 0.000 | 0.000 | 0.053 | 0.000 | 0.050 | 0.000 |
|  | *H_e_* | 0.049 | 0.049 | 0.000 | 0.000 | 0.000 | 0.000 | 0.049 | 0.000 | 0.000 | 0.000 | 0.000 | 0.000 | 0.000 | 0.000 | 0.000 | 0.000 | 0.000 | 0.051 | 0.000 | 0.049 | 0.000 |
|  | *F_is_* | -0.026 | -0.026 | #N/A | #N/A | #N/A | #N/A | -0.026 | #N/A | #N/A | #N/A | #N/A | #N/A | #N/A | #N/A | #N/A | #N/A | #N/A | -0.027 | #N/A | -0.026 | #N/A |
| **MI-locust_2012** | *H_o_* | 0.000 | 0.000 | 0.000 | 0.000 | 0.000 | 0.000 | 0.000 | 0.000 | 0.000 | 0.000 | 0.000 | 0.000 | 0.000 | 0.000 | 0.000 | 0.000 | 0.000 | 0.000 | 0.000 | 0.000 | 0.000 |
|  | *H_e_* | 0.000 | 0.000 | 0.000 | 0.000 | 0.000 | 0.000 | 0.000 | 0.000 | 0.000 | 0.000 | 0.000 | 0.000 | 0.000 | 0.000 | 0.000 | 0.000 | 0.000 | 0.000 | 0.000 | 0.000 | 0.000 |
|  | *F_is_* | #N/A | #N/A | #N/A | #N/A | #N/A | #N/A | #N/A | #N/A | #N/A | #N/A | #N/A | #N/A | #N/A | #N/A | #N/A | #N/A | #N/A | #N/A | #N/A | #N/A | #N/A |
| **625&231_2012** | *H_o_* | 0.000 | 0.000 | 0.000 | 0.000 | 0.000 | 0.000 | 0.000 | 0.000 | 0.000 | 0.000 | 0.000 | 0.000 | 0.000 | 0.000 | 0.000 | 0.000 | 0.000 | 0.000 | 0.000 | 0.000 | 0.000 |
|  | *H_e_* | 0.000 | 0.000 | 0.000 | 0.000 | 0.000 | 0.000 | 0.000 | 0.000 | 0.000 | 0.000 | 0.000 | 0.000 | 0.000 | 0.000 | 0.000 | 0.000 | 0.000 | 0.000 | 0.000 | 0.000 | 0.000 |
|  | *F_is_* | #N/A | #N/A | #N/A | #N/A | #N/A | #N/A | #N/A | #N/A | #N/A | #N/A | #N/A | #N/A | #N/A | #N/A | #N/A | #N/A | #N/A | #N/A | #N/A | #N/A | #N/A |
| **SR16&280W_2012** | *H_o_* | 0.263 | 0.263 | 0.000 | 0.211 | 0.000 | 0.000 | 0.263 | 0.000 | 0.000 | 0.000 | 0.000 | 0.000 | 0.000 | 0.000 | 0.000 | 0.000 | 0.000 | 0.263 | 0.000 | 0.263 | 0.000 |
|  | *H_e_* | 0.229 | 0.229 | 0.000 | 0.188 | 0.000 | 0.000 | 0.229 | 0.000 | 0.000 | 0.000 | 0.000 | 0.000 | 0.000 | 0.000 | 0.000 | 0.000 | 0.000 | 0.229 | 0.000 | 0.229 | 0.000 |
|  | *F_is_* | -0.152 | -0.152 | #N/A | -0.118 | #N/A | #N/A | -0.152 | #N/A | #N/A | #N/A | #N/A | #N/A | #N/A | #N/A | #N/A | #N/A | #N/A | -0.152 | #N/A | -0.152 | #N/A |
| **Hwy43&I-65_2012** | *H_o_* | 0.000 | 0.000 | 0.000 | 0.000 | 0.000 | 0.000 | 0.000 | 0.000 | 0.000 | 0.000 | 0.000 | 0.000 | 0.000 | 0.000 | 0.000 | 0.000 | 0.000 | 0.000 | 0.000 | 0.000 | 0.000 |
|  | *H_e_* | 0.000 | 0.000 | 0.000 | 0.000 | 0.000 | 0.000 | 0.000 | 0.000 | 0.000 | 0.000 | 0.000 | 0.000 | 0.000 | 0.000 | 0.000 | 0.000 | 0.000 | 0.000 | 0.000 | 0.000 | 0.000 |
|  | *F_is_* | #N/A | #N/A | #N/A | #N/A | #N/A | #N/A | #N/A | #N/A | #N/A | #N/A | #N/A | #N/A | #N/A | #N/A | #N/A | #N/A | #N/A | #N/A | #N/A | #N/A | #N/A |
| **Meridian&218E_2012** | *H_o_* | 0.000 | 0.000 | 0.000 | 0.000 | 0.000 | 0.000 | 0.000 | 0.000 | 0.000 | 0.000 | 0.000 | 0.000 | 0.000 | 0.000 | 0.000 | 0.000 | 0.000 | 0.000 | 0.000 | 0.000 | 0.000 |
|  | *H_e_* | 0.000 | 0.000 | 0.000 | 0.000 | 0.000 | 0.000 | 0.000 | 0.000 | 0.000 | 0.000 | 0.000 | 0.000 | 0.000 | 0.000 | 0.000 | 0.000 | 0.000 | 0.000 | 0.000 | 0.000 | 0.000 |
|  | *F_is_* | #N/A | #N/A | #N/A | #N/A | #N/A | #N/A | #N/A | #N/A | #N/A | #N/A | #N/A | #N/A | #N/A | #N/A | #N/A | #N/A | #N/A | #N/A | #N/A | #N/A | #N/A |
| **Hwy52&Walnut_2012** | *H_o_* | 0.059 | 0.063 | 0.059 | 0.059 | 0.059 | 0.059 | 0.059 | 0.059 | 0.063 | 0.059 | 0.059 | 0.059 | 0.059 | 0.059 | 0.059 | 0.059 | 0.067 | 0.059 | 0.059 | 0.059 | 0.059 |
|  | *H_e_* | 0.057 | 0.061 | 0.057 | 0.057 | 0.057 | 0.057 | 0.057 | 0.057 | 0.061 | 0.057 | 0.057 | 0.057 | 0.057 | 0.057 | 0.057 | 0.057 | 0.064 | 0.057 | 0.057 | 0.057 | 0.057 |
|  | *F_is_* | -0.030 | -0.032 | -0.030 | -0.030 | -0.030 | -0.030 | -0.030 | -0.030 | -0.032 | -0.030 | -0.030 | -0.030 | -0.030 | -0.030 | -0.030 | -0.030 | -0.034 | -0.030 | -0.030 | -0.030 | -0.030 |
| **IN-OH_2012** | *H_o_* | 0.000 | 0.000 | 0.000 | 0.000 | 0.000 | 0.000 | 0.000 | 0.000 | 0.000 | 0.000 | 0.000 | 0.000 | 0.000 | 0.000 | 0.000 | 0.000 | 0.000 | 0.000 | 0.000 | 0.000 | 0.000 |
|  | *H_e_* | 0.000 | 0.000 | 0.000 | 0.000 | 0.000 | 0.000 | 0.000 | 0.000 | 0.000 | 0.000 | 0.000 | 0.000 | 0.000 | 0.000 | 0.000 | 0.000 | 0.000 | 0.000 | 0.000 | 0.000 | 0.000 |
|  | *F_is_* | #N/A | #N/A | #N/A | #N/A | #N/A | #N/A | #N/A | #N/A | #N/A | #N/A | #N/A | #N/A | #N/A | #N/A | #N/A | #N/A | #N/A | #N/A | #N/A | #N/A | #N/A |
| **MI-clover_2012** | *H_o_* | 1.000 | 1.000 | 1.000 | 1.000 | 1.000 | 1.000 | 1.000 | 1.000 | 1.000 | 1.000 | 1.000 | 1.000 | 1.000 | 1.000 | 1.000 | 1.000 | 1.000 | 1.000 | 1.000 | 1.000 | 1.000 |
|  | *H_e_* | 0.500 | 0.500 | 0.500 | 0.500 | 0.500 | 0.500 | 0.500 | 0.500 | 0.500 | 0.500 | 0.500 | 0.500 | 0.500 | 0.500 | 0.500 | 0.500 | 0.500 | 0.500 | 0.500 | 0.500 | 0.500 |
|  | *F_is_* | **-1.000** | **-1.000** | **-1.000** | **-1.000** | **-1.000** | **-1.000** | **-1.000** | **-1.000** | **-1.000** | **-1.000** | **-1.000** | **-1.000** | **-1.000** | **-1.000** | **-1.000** | **-1.000** | **-1.000** | **-1.000** | **-1.000** | **-1.000** | **-1.000** |
| **Chelsea_2012** | *H_o_* | 1.000 | 1.000 | 1.000 | 1.000 | 1.000 | 1.000 | 1.000 | 1.000 | 1.000 | 1.000 | 1.000 | 1.000 | 1.000 | 1.000 | 1.000 | 1.000 | 1.000 | 1.000 | 1.000 | 1.000 | 1.000 |
|  | *H_e_* | 0.500 | 0.500 | 0.500 | 0.500 | 0.500 | 0.500 | 0.500 | 0.500 | 0.500 | 0.500 | 0.500 | 0.500 | 0.500 | 0.500 | 0.500 | 0.500 | 0.500 | 0.500 | 0.500 | 0.500 | 0.500 |
|  | *F_is_* | **-1.000** | **-1.000** | **-1.000** | **-1.000** | **-1.000** | **-1.000** | **-1.000** | **-1.000** | **-1.000** | **-1.000** | **-1.000** | **-1.000** | **-1.000** | **-1.000** | **-1.000** | **-1.000** | **-1.000** | **-1.000** | **-1.000** | **-1.000** | **-1.000** |
| **BuckCreek2-3_2012** | *H_o_* | 0.667 | 0.667 | 0.667 | **0.933** | 0.667 | 0.667 | **0.733** | **0.867** | 0.667 | 0.667 | 0.733 | **0.786** | 0.667 | 0.667 | 0.667 | 0.667 | **0.733** | **0.867** | 0.667 | 0.667 | 0.667 |
|  | *H_e_* | 0.444 | 0.444 | 0.444 | 0.498 | 0.444 | 0.444 | 0.464 | 0.500 | 0.444 | 0.444 | 0.491 | 0.477 | 0.444 | 0.444 | 0.498 | 0.444 | 0.464 | 0.491 | 0.498 | 0.444 | 0.444 |
|  | *F_is_* | -0.500 | -0.500 | -0.500 | -0.875 | -0.500 | -0.500 | -0.579 | -0.733 | -0.500 | -0.500 | -0.493 | -0.647 | -0.500 | -0.500 | -0.339 | -0.500 | -0.579 | -0.765 | -0.339 | -0.500 | -0.500 |
| **Otterbein_2012** | *H_o_* | 1.000 | 1.000 | 1.000 | 1.000 | 1.000 | 1.000 | 1.000 | 1.000 | 1.000 | 1.000 | 1.000 | 1.000 | 1.000 | 1.000 | 1.000 | 1.000 | 1.000 | 1.000 | 1.000 | 1.000 | 1.000 |
|  | *H_e_* | 0.500 | 0.500 | 0.500 | 0.500 | 0.500 | 0.500 | 0.500 | 0.500 | 0.500 | 0.500 | 0.500 | 0.500 | 0.500 | 0.500 | 0.500 | 0.500 | 0.500 | 0.500 | 0.500 | 0.500 | 0.500 |
|  | *F_is_* | **-1.000** | **-1.000** | **-1.000** | **-1.000** | **-1.000** | **-1.000** | **-1.000** | **-1.000** | **-1.000** | **-1.000** | **-1.000** | **-1.000** | **-1.000** | **-1.000** | **-1.000** | **-1.000** | **-1.000** | **-1.000** | **-1.000** | **-1.000** | **-1.000** |
| **USDA-Madison_2012** | *H_o_* | 0.000 | 0.000 | 0.000 | 0.000 | 0.000 | 0.000 | 0.000 | 0.000 | 0.000 | 0.000 | 0.000 | 0.000 | 0.000 | 0.000 | 0.000 | 0.000 | 0.000 | 0.000 | 0.000 | 0.000 | 0.000 |
|  | *H_e_* | 0.000 | 0.000 | 0.000 | 0.000 | 0.000 | 0.000 | 0.000 | 0.000 | 0.000 | 0.000 | 0.000 | 0.000 | 0.000 | 0.000 | 0.000 | 0.000 | 0.000 | 0.000 | 0.000 | 0.000 | 0.000 |
|  | *F_is_* | #N/A | #N/A | #N/A | #N/A | #N/A | #N/A | #N/A | #N/A | #N/A | #N/A | #N/A | #N/A | #N/A | #N/A | #N/A | #N/A | #N/A | #N/A | #N/A | #N/A | #N/A |
| **WI_2012** | *H_o_* | 1.000 | 1.000 | 1.000 | 1.000 | 1.000 | 1.000 | 1.000 | 1.000 | 1.000 | 1.000 | 1.000 | 1.000 | 1.000 | 1.000 | 1.000 | 1.000 | 1.000 | 1.000 | 1.000 | 1.000 | 1.000 |
|  | *H_e_* | 0.500 | 0.500 | 0.500 | 0.500 | 0.500 | 0.500 | 0.500 | 0.500 | 0.500 | 0.500 | 0.500 | 0.500 | 0.500 | 0.500 | 0.500 | 0.500 | 0.500 | 0.500 | 0.500 | 0.500 | 0.500 |
|  | *F_is_* | **-1.000** | **-1.000** | **-1.000** | **-1.000** | **-1.000** | **-1.000** | **-1.000** | **-1.000** | **-1.000** | **-1.000** | **-1.000** | **-1.000** | **-1.000** | **-1.000** | **-1.000** | **-1.000** | **-1.000** | **-1.000** | **-1.000** | **-1.000** | **-1.000** |
| **IL_2012** | *H_o_* | 1.000 | 1.000 | 1.000 | 1.000 | 1.000 | 1.000 | 1.000 | 1.000 | 1.000 | 1.000 | 1.000 | 1.000 | 1.000 | 1.000 | 1.000 | 1.000 | 1.000 | 1.000 | 1.000 | 1.000 | 1.000 |
|  | *H_e_* | 0.500 | 0.500 | 0.500 | 0.500 | 0.500 | 0.500 | 0.500 | 0.500 | 0.500 | 0.500 | 0.500 | 0.500 | 0.500 | 0.500 | 0.500 | 0.500 | 0.500 | 0.500 | 0.500 | 0.500 | 0.500 |
|  | *F_is_* | **-1.000** | **-1.000** | **-1.000** | **-1.000** | **-1.000** | **-1.000** | **-1.000** | **-1.000** | **-1.000** | **-1.000** | **-1.000** | **-1.000** | **-1.000** | **-1.000** | **-1.000** | **-1.000** | **-1.000** | **-1.000** | **-1.000** | **-1.000** | **-1.000** |
| **KY_2012** | *H_o_* | 1.000 | 1.000 | 1.000 | 1.000 | 1.000 | 1.000 | 1.000 | 1.000 | 1.000 | 1.000 | 1.000 | 1.000 | 1.000 | 1.000 | 1.000 | 1.000 | 1.000 | 1.000 | 1.000 | 1.000 | 1.000 |
|  | *H_e_* | 0.500 | 0.500 | 0.500 | 0.500 | 0.500 | 0.500 | 0.500 | 0.500 | 0.500 | 0.500 | 0.500 | 0.500 | 0.500 | 0.500 | 0.500 | 0.500 | 0.500 | 0.500 | 0.500 | 0.500 | 0.500 |
|  | *F_is_* | **-1.000** | **-1.000** | **-1.000** | **-1.000** | **-1.000** | **-1.000** | **-1.000** | **-1.000** | **-1.000** | **-1.000** | **-1.000** | **-1.000** | **-1.000** | **-1.000** | **-1.000** | **-1.000** | **-1.000** | **-1.000** | **-1.000** | **-1.000** | **-1.000** |
| **TPAC_2012** | *H_o_* | 1.000 | 1.000 | 1.000 | 1.000 | 1.000 | 1.000 | 1.000 | 1.000 | 1.000 | 1.000 | 1.000 | 1.000 | 1.000 | 1.000 | 1.000 | 1.000 | 1.000 | 1.000 | 1.000 | 1.000 | 1.000 |
|  | *H_e_* | 0.500 | 0.500 | 0.500 | 0.500 | 0.500 | 0.500 | 0.500 | 0.500 | 0.500 | 0.500 | 0.500 | 0.500 | 0.500 | 0.500 | 0.500 | 0.500 | 0.500 | 0.500 | 0.500 | 0.500 | 0.500 |
|  | *F_is_* | **-1.000** | **-1.000** | **-1.000** | **-1.000** | **-1.000** | **-1.000** | **-1.000** | **-1.000** | **-1.000** | **-1.000** | **-1.000** | **-1.000** | **-1.000** | **-1.000** | **-1.000** | **-1.000** | **-1.000** | **-1.000** | **-1.000** | **-1.000** | **-1.000** |
| **WieseN_2012** | *H_o_* | 1.000 | 1.000 | 1.000 | 1.000 | 1.000 | 1.000 | 1.000 | 1.000 | 1.000 | 1.000 | 1.000 | 1.000 | 1.000 | 1.000 | 1.000 | 1.000 | 1.000 | 1.000 | 1.000 | 1.000 | 1.000 |
|  | *H_e_* | 0.500 | 0.500 | 0.500 | 0.500 | 0.500 | 0.500 | 0.500 | 0.500 | 0.500 | 0.500 | 0.500 | 0.500 | 0.500 | 0.500 | 0.500 | 0.500 | 0.500 | 0.500 | 0.500 | 0.500 | 0.500 |
|  | *F_is_* | **-1.000** | **-1.000** | **-1.000** | **-1.000** | **-1.000** | **-1.000** | **-1.000** | **-1.000** | **-1.000** | **-1.000** | **-1.000** | **-1.000** | **-1.000** | **-1.000** | **-1.000** | **-1.000** | **-1.000** | **-1.000** | **-1.000** | **-1.000** | **-1.000** |
| **Cole7_2012** | *H_o_* | 1.000 | 1.000 | 1.000 | 1.000 | 1.000 | 1.000 | 1.000 | 1.000 | 1.000 | 1.000 | 1.000 | 1.000 | 1.000 | 1.000 | 1.000 | 1.000 | 1.000 | 1.000 | 1.000 | 1.000 | 1.000 |
|  | *H_e_* | 0.500 | 0.500 | 0.500 | 0.500 | 0.500 | 0.500 | 0.500 | 0.500 | 0.500 | 0.500 | 0.500 | 0.500 | 0.500 | 0.500 | 0.500 | 0.500 | 0.500 | 0.500 | 0.500 | 0.500 | 0.500 |
|  | *F_is_* | **-1.000** | **-1.000** | **-1.000** | **-1.000** | **-1.000** | **-1.000** | **-1.000** | **-1.000** | **-1.000** | **-1.000** | **-1.000** | **-1.000** | **-1.000** | **-1.000** | **-1.000** | **-1.000** | **-1.000** | **-1.000** | **-1.000** | **-1.000** | **-1.000** |
| **Buck1_2012** | *H_o_* | 1.000 | 1.000 | 1.000 | 1.000 | 1.000 | 1.000 | 1.000 | 1.000 | 1.000 | 1.000 | 1.000 | 1.000 | 1.000 | 1.000 | 1.000 | 1.000 | 1.000 | 1.000 | 1.000 | 1.000 | 1.000 |
|  | *H_e_* | 0.500 | 0.500 | 0.500 | 0.500 | 0.500 | 0.500 | 0.500 | 0.500 | 0.500 | 0.500 | 0.500 | 0.500 | 0.500 | 0.500 | 0.500 | 0.500 | 0.500 | 0.500 | 0.500 | 0.500 | 0.500 |
|  | *F_is_* | **-1.000** | **-1.000** | **-1.000** | **-1.000** | **-1.000** | **-1.000** | **-1.000** | **-1.000** | **-1.000** | **-1.000** | **-1.000** | **-1.000** | **-1.000** | **-1.000** | **-1.000** | **-1.000** | **-1.000** | **-1.000** | **-1.000** | **-1.000** | **-1.000** |
| **Crosby_2012** | *H_o_* | 1.000 | 1.000 | 1.000 | 1.000 | 1.000 | 1.000 | 1.000 | 1.000 | 1.000 | 1.000 | 1.000 | 1.000 | 1.000 | 1.000 | 1.000 | 1.000 | 1.000 | 1.000 | 1.000 | 1.000 | 1.000 |
|  | *H_e_* | 0.500 | 0.500 | 0.500 | 0.500 | 0.500 | 0.500 | 0.500 | 0.500 | 0.500 | 0.500 | 0.500 | 0.500 | 0.500 | 0.500 | 0.500 | 0.500 | 0.500 | 0.500 | 0.500 | 0.500 | 0.500 |
|  | *F_is_* | **-1.000** | **-1.000** | **-1.000** | **-1.000** | **-1.000** | **-1.000** | **-1.000** | **-1.000** | **-1.000** | **-1.000** | **-1.000** | **-1.000** | **-1.000** | **-1.000** | **-1.000** | **-1.000** | **-1.000** | **-1.000** | **-1.000** | **-1.000** | **-1.000** |
| **MI_2012** | *H_o_* | 1.000 | 1.000 | 1.000 | 1.000 | 1.000 | 1.000 | 1.000 | 1.000 | 1.000 | 1.000 | 1.000 | 1.000 | 1.000 | 1.000 | 1.000 | 1.000 | 1.000 | 1.000 | 1.000 | 1.000 | 1.000 |
|  | *H_e_* | 0.500 | 0.500 | 0.500 | 0.500 | 0.500 | 0.500 | 0.500 | 0.500 | 0.500 | 0.500 | 0.500 | 0.500 | 0.500 | 0.500 | 0.500 | 0.500 | 0.500 | 0.500 | 0.500 | 0.500 | 0.500 |
|  | *F_is_* | **-1.000** | **-1.000** | **-1.000** | **-1.000** | **-1.000** | **-1.000** | **-1.000** | **-1.000** | **-1.000** | **-1.000** | **-1.000** | **-1.000** | **-1.000** | **-1.000** | **-1.000** | **-1.000** | **-1.000** | **-1.000** | **-1.000** | **-1.000** | **-1.000** |
| **pan_2013** | *H_o_* | 0.200 | 0.220 | **0.195** | 0.711 | 0.220 | 0.195 | 0.390 | 0.450 | 0.195 | 0.175 | **0.390** | 0.564 | 0.195 | 0.195 | 0.400 | 0.205 | 0.293 | 0.561 | 0.400 | 0.244 | 0.175 |
|  | *H_e_* | 0.180 | 0.195 | 0.176 | 0.483 | 0.195 | 0.176 | 0.343 | 0.495 | 0.176 | 0.160 | 0.500 | 0.405 | 0.176 | 0.176 | 0.495 | 0.184 | 0.250 | 0.470 | 0.480 | 0.214 | 0.160 |
|  | *F_is_* | -0.111 | -0.123 | -0.108 | -0.471 | -0.123 | -0.108 | -0.139 | 0.091 | -0.108 | -0.096 | 0.219 | -0.393 | -0.108 | -0.108 | 0.192 | -0.114 | -0.171 | -0.193 | 0.167 | -0.139 | -0.096 |
| **MIbl** | *H_o_* | 0.000 | 0.000 | 0.000 | 0.889 | 0.000 | 0.000 | 0.000 | 0.000 | 0.000 | 0.000 | 0.000 | 0.000 | 0.000 | 0.000 | 0.000 | 0.000 | 0.000 | 0.000 | 0.000 | 0.000 | 0.000 |
|  | *H_e_* | 0.000 | 0.000 | 0.000 | 0.494 | 0.000 | 0.000 | 0.000 | 0.000 | 0.000 | 0.000 | 0.000 | 0.000 | 0.000 | 0.000 | 0.000 | 0.000 | 0.000 | 0.000 | 0.000 | 0.000 | 0.000 |
|  | *F_is_* | #N/A | #N/A | #N/A | **-0.800** | #N/A | #N/A | #N/A | #N/A | #N/A | #N/A | #N/A | #N/A | #N/A | #N/A | #N/A | #N/A | #N/A | #N/A | #N/A | #N/A | #N/A |
| **OHbl** | *H_o_* | 0.778 | 0.833 | 0.778 | 0.889 | 0.875 | 0.765 | 0.889 | 0.833 | 0.778 | 0.765 | 0.889 | 0.833 | 0.778 | 0.778 | 0.944 | 0.778 | 0.778 | 0.833 | 0.882 | 0.778 | 0.778 |
|  | *H_e_* | 0.475 | 0.486 | 0.475 | 0.494 | 0.492 | 0.472 | 0.494 | 0.486 | 0.475 | 0.472 | 0.494 | 0.486 | 0.475 | 0.475 | 0.498 | 0.475 | 0.475 | 0.486 | 0.493 | 0.475 | 0.475 |
|  | *F_is_* | **-0.636** | **-0.714** | **-0.636** | **-0.800** | **-0.778** | **-0.619** | **-0.800** | **-0.714** | **-0.636** | **-0.619** | **-0.800** | **-0.714** | **-0.636** | **-0.636** | **-0.895** | **-0.636** | **-0.636** | **-0.714** | **-0.789** | **-0.636** | **-0.636** |
| **KYbl** | *H_o_* | 0.000 | 0.000 | 0.000 | **0.900** | 0.000 | 0.000 | 0.000 | 0.000 | 0.000 | 0.000 | 0.000 | 0.000 | 0.000 | 0.000 | 0.000 | 0.000 | 0.000 | 0.000 | 0.000 | 0.000 | 0.000 |
|  | *H_e_* | 0.000 | 0.000 | 0.000 | 0.495 | 0.000 | 0.000 | 0.000 | 0.000 | 0.000 | 0.000 | 0.000 | 0.000 | 0.000 | 0.000 | 0.000 | 0.000 | 0.000 | 0.000 | 0.000 | 0.000 | 0.000 |
|  | *F_is_* | #N/A | #N/A | #N/A | -0.818 | #N/A | #N/A | #N/A | #N/A | #N/A | #N/A | #N/A | #N/A | #N/A | #N/A | #N/A | #N/A | #N/A | #N/A | #N/A | #N/A | #N/A |
| **wcINbl** | *H_o_* | 0.000 | 0.000 | 0.000 | **0.056** | 0.000 | 0.000 | 0.000 | 0.000 | 0.000 | 0.000 | 0.000 | 0.000 | 0.000 | 0.000 | 0.000 | 0.000 | 0.000 | 0.000 | 0.000 | 0.000 | 0.000 |
|  | *H_e_* | 0.000 | 0.000 | 0.000 | 0.054 | 0.000 | 0.000 | 0.000 | 0.000 | 0.000 | 0.000 | 0.000 | 0.000 | 0.000 | 0.000 | 0.000 | 0.000 | 0.000 | 0.000 | 0.000 | 0.000 | 0.000 |
|  | *F_is_* | #N/A | #N/A | #N/A | -0.029 | #N/A | #N/A | #N/A | #N/A | #N/A | #N/A | #N/A | #N/A | #N/A | #N/A | #N/A | #N/A | #N/A | #N/A | #N/A | #N/A | #N/A |
| **nearMTbl3** | *H_o_* | 0.000 | 0.000 | 0.000 | 0.000 | 0.000 | 0.000 | 0.000 | 0.000 | 0.000 | 0.000 | 0.000 | 0.000 | 0.000 | 0.000 | 0.000 | 0.000 | 0.000 | 0.000 | 0.000 | 0.000 | 0.000 |
|  | *H_e_* | 0.000 | 0.000 | 0.000 | 0.000 | 0.000 | 0.000 | 0.000 | 0.000 | 0.000 | 0.000 | 0.000 | 0.000 | 0.000 | 0.000 | 0.000 | 0.000 | 0.000 | 0.000 | 0.000 | 0.000 | 0.000 |
|  | *F_is_* | #N/A | #N/A | #N/A | #N/A | #N/A | #N/A | #N/A | #N/A | #N/A | #N/A | #N/A | #N/A | #N/A | #N/A | #N/A | #N/A | #N/A | #N/A | #N/A | #N/A | #N/A |
| **CarolCoIN** | *H_o_* | 0.000 | 0.000 | 0.000 | 0.000 | 0.000 | 0.000 | 0.000 | 0.000 | 0.000 | 0.000 | 0.000 | 0.000 | 0.000 | 0.000 | 0.000 | 0.000 | 0.000 | 0.000 | 0.000 | 0.000 | 0.000 |
|  | *H_e_* | 0.000 | 0.000 | 0.000 | 0.000 | 0.000 | 0.000 | 0.000 | 0.000 | 0.000 | 0.000 | 0.000 | 0.000 | 0.000 | 0.000 | 0.000 | 0.000 | 0.000 | 0.000 | 0.000 | 0.000 | 0.000 |
|  | *F_is_* | #N/A | #N/A | #N/A | #N/A | #N/A | #N/A | #N/A | #N/A | #N/A | #N/A | #N/A | #N/A | #N/A | #N/A | #N/A | #N/A | #N/A | #N/A | #N/A | #N/A | #N/A |
| **nearOKbl** | *H_o_* | 0.000 | 0.000 | 0.000 | **0.850** | 0.000 | 0.000 | 0.000 | 0.000 | 0.000 | 0.000 | 0.000 | 0.000 | 0.000 | 0.000 | 0.000 | 0.000 | 0.000 | 0.000 | 0.000 | 0.000 | 0.000 |
|  | *H_e_* | 0.000 | 0.000 | 0.000 | 0.489 | 0.000 | 0.000 | 0.000 | 0.000 | 0.000 | 0.000 | 0.000 | 0.000 | 0.000 | 0.000 | 0.000 | 0.000 | 0.000 | 0.000 | 0.000 | 0.000 | 0.000 |
|  | *F_is_* | #N/A | #N/A | #N/A | -0.739 | #N/A | #N/A | #N/A | #N/A | #N/A | #N/A | #N/A | #N/A | #N/A | #N/A | #N/A | #N/A | #N/A | #N/A | #N/A | #N/A | #N/A |
| **btwnMObl** | *H_o_* | 0.059 | 0.059 | 0.118 | 0.500 | 0.059 | 0.059 | 0.059 | 0.000 | 0.059 | 0.063 | 0.059 | 0.059 | 0.000 | 0.059 | 0.059 | 0.059 | 0.059 | 0.000 | 0.059 | 0.059 | 0.059 |
|  | *H_e_* | 0.057 | 0.057 | 0.111 | 0.375 | 0.057 | 0.057 | 0.057 | 0.000 | 0.057 | 0.061 | 0.057 | 0.057 | 0.000 | 0.057 | 0.057 | 0.057 | 0.057 | 0.000 | 0.057 | 0.057 | 0.057 |
|  | *F_is_* | -0.030 | -0.030 | -0.063 | -0.333 | -0.030 | -0.030 | -0.030 | #N/A | -0.030 | -0.032 | -0.030 | -0.030 | #N/A | -0.030 | -0.030 | -0.030 | -0.030 | #N/A | -0.030 | -0.030 | -0.030 |
| **KnoxIL** | *H_o_* | 1.000 | 1.000 | 1.000 | 1.000 | 1.000 | 1.000 | 1.000 | 1.000 | 1.000 | 1.000 | 1.000 | 1.000 | 1.000 | 1.000 | 1.000 | 1.000 | 1.000 | 1.000 | 1.000 | 1.000 | 1.000 |
|  | *H_e_* | 0.500 | 0.500 | 0.500 | 0.500 | 0.500 | 0.500 | 0.500 | 0.500 | 0.500 | 0.500 | 0.500 | 0.500 | 0.500 | 0.500 | 0.500 | 0.500 | 0.500 | 0.500 | 0.500 | 0.500 | 0.500 |
|  | *F_is_* | **-1.000** | **-1.000** | **-1.000** | **-1.000** | **-1.000** | **-1.000** | **-1.000** | **-1.000** | **-1.000** | **-1.000** | **-1.000** | **-1.000** | **-1.000** | **-1.000** | **-1.000** | **-1.000** | **-1.000** | **-1.000** | **-1.000** | **-1.000** | **-1.000** |
| **ArgyleWI** | *H_o_* | 0.950 | 0.950 | 0.950 | 0.950 | 0.950 | 0.950 | 1.000 | 1.000 | 0.950 | 0.947 | 0.950 | 1.000 | 0.950 | 0.950 | 1.000 | 0.950 | 1.000 | 0.950 | 1.000 | 0.950 | 0.950 |
|  | *H_e_* | 0.499 | 0.499 | 0.499 | 0.499 | 0.499 | 0.499 | 0.500 | 0.500 | 0.499 | 0.499 | 0.499 | 0.500 | 0.499 | 0.499 | 0.500 | 0.499 | 0.500 | 0.499 | 0.500 | 0.499 | 0.499 |
|  | *F_is_* | **-0.905** | **-0.905** | **-0.905** | **-0.905** | **-0.905** | **-0.905** | **-1.000** | **-1.000** | **-0.905** | **-0.900** | **-0.905** | **-1.000** | **-0.905** | **-0.905** | **-1.000** | **-0.905** | **-1.000** | **-0.905** | **-1.000** | **-0.905** | **-0.905** |
| **PawPawMI** | *H_o_* | 1.000 | 1.000 | 1.000 | 1.000 | 1.000 | 1.000 | 1.000 | 1.000 | 1.000 | 1.000 | 1.000 | 1.000 | 1.000 | 1.000 | 1.000 | 1.000 | 1.000 | 1.000 | 1.000 | 1.000 | 1.000 |
|  | *H_e_* | 0.500 | 0.500 | 0.500 | 0.500 | 0.500 | 0.500 | 0.500 | 0.500 | 0.500 | 0.500 | 0.500 | 0.500 | 0.500 | 0.500 | 0.500 | 0.500 | 0.500 | 0.500 | 0.500 | 0.500 | 0.500 |
|  | *F_is_* | **-1.000** | **-1.000** | **-1.000** | **-1.000** | **-1.000** | **-1.000** | **-1.000** | **-1.000** | **-1.000** | **-1.000** | **-1.000** | **-1.000** | **-1.000** | **-1.000** | **-1.000** | **-1.000** | **-1.000** | **-1.000** | **-1.000** | **-1.000** | **-1.000** |
| **sIN** | *H_o_* | 0.933 | 0.933 | 0.933 | 0.933 | 0.933 | 0.933 | 0.933 | 0.933 | 0.933 | 1.000 | 1.000 | 1.000 | 0.933 | 0.933 | 0.933 | 0.933 | 0.933 | 0.933 | 0.929 | 0.933 | 0.933 |
|  | *H_e_* | 0.498 | 0.498 | 0.498 | 0.498 | 0.498 | 0.498 | 0.498 | 0.498 | 0.498 | 0.500 | 0.500 | 0.500 | 0.498 | 0.498 | 0.498 | 0.498 | 0.498 | 0.498 | 0.497 | 0.498 | 0.498 |
|  | *F_is_* | **-0.875** | **-0.875** | **-0.875** | **-0.875** | **-0.875** | **-0.875** | **-0.875** | **-0.875** | **-0.875** | **-1.000** | **-1.000** | **-1.000** | **-0.875** | **-0.875** | **-0.875** | **-0.875** | **-0.875** | **-0.875** | **-0.867** | **-0.875** | **-0.875** |
| **wcIN** | *H_o_* | 0.947 | 0.947 | 0.947 | 1.000 | 0.950 | 0.950 | 0.950 | 0.950 | 0.950 | 0.950 | 0.947 | 0.950 | 0.950 | 0.950 | 0.950 | 0.950 | 0.947 | 0.950 | 0.950 | 0.950 | 0.950 |
|  | *H_e_* | 0.499 | 0.499 | 0.499 | 0.500 | 0.499 | 0.499 | 0.499 | 0.499 | 0.499 | 0.499 | 0.499 | 0.499 | 0.499 | 0.499 | 0.499 | 0.499 | 0.499 | 0.499 | 0.499 | 0.499 | 0.499 |
|  | *F_is_* | **-0.900** | **-0.900** | **-0.900** | **-1.000** | **-0.905** | **-0.905** | **-0.905** | **-0.905** | **-0.905** | **-0.905** | **-0.900** | **-0.905** | **-0.905** | **-0.905** | **-0.905** | **-0.905** | **-0.900** | **-0.905** | **-0.905** | **-0.905** | **-0.905** |
| **nearMT** | *H_o_* | 1.000 | 1.000 | 1.000 | 1.000 | 1.000 | 1.000 | 1.000 | 1.000 | 1.000 | 1.000 | 1.000 | 1.000 | 1.000 | 1.000 | 1.000 | 1.000 | 1.000 | 1.000 | 1.000 | 1.000 | 1.000 |
|  | *H_e_* | 0.500 | 0.500 | 0.500 | 0.500 | 0.500 | 0.500 | 0.500 | 0.500 | 0.500 | 0.500 | 0.500 | 0.500 | 0.500 | 0.500 | 0.500 | 0.500 | 0.500 | 0.500 | 0.500 | 0.500 | 0.500 |
|  | *F_is_* | **-1.000** | **-1.000** | **-1.000** | **-1.000** | **-1.000** | **-1.000** | **-1.000** | **-1.000** | **-1.000** | **-1.000** | **-1.000** | **-1.000** | **-1.000** | **-1.000** | **-1.000** | **-1.000** | **-1.000** | **-1.000** | **-1.000** | **-1.000** | **-1.000** |
| **nearM** | *H_o_* | 0.947 | 0.950 | 0.900 | 0.950 | 0.900 | 0.895 | 0.947 | 0.900 | 0.900 | 0.900 | 0.950 | 0.950 | 0.900 | 0.900 | 0.950 | 0.900 | 0.900 | 1.000 | 0.900 | 0.947 | 0.900 |
|  | *H_e_* | 0.499 | 0.499 | 0.495 | 0.499 | 0.495 | 0.494 | 0.499 | 0.500 | 0.495 | 0.495 | 0.499 | 0.499 | 0.495 | 0.495 | 0.499 | 0.495 | 0.495 | 0.500 | 0.500 | 0.499 | 0.495 |
|  | *F_is_* | **-0.900** | **-0.905** | **-0.818** | **-0.905** | **-0.818** | **-0.810** | **-0.900** | **-0.800** | **-0.818** | **-0.818** | **-0.905** | **-0.905** | **-0.818** | **-0.818** | **-0.905** | **-0.818** | **-0.818** | **-1.000** | **-0.800** | **-0.900** | **-0.818** |
| **nearOK10** | *H_o_* | 1.000 | 1.000 | 1.000 | 1.000 | 1.000 | 1.000 | 1.000 | 1.000 | 1.000 | 1.000 | 1.000 | 1.000 | 1.000 | 1.000 | 1.000 | 1.000 | 1.000 | 1.000 | 1.000 | 1.000 | 1.000 |
|  | *H_e_* | 0.500 | 0.500 | 0.500 | 0.500 | 0.500 | 0.500 | 0.500 | 0.500 | 0.500 | 0.500 | 0.500 | 0.500 | 0.500 | 0.500 | 0.500 | 0.500 | 0.500 | 0.500 | 0.500 | 0.500 | 0.500 |
|  | *F_is_* | **-1.000** | **-1.000** | **-1.000** | **-1.000** | **-1.000** | **-1.000** | **-1.000** | **-1.000** | **-1.000** | **-1.000** | **-1.000** | **-1.000** | **-1.000** | **-1.000** | **-1.000** | **-1.000** | **-1.000** | **-1.000** | **-1.000** | **-1.000** | **-1.000** |
| **nearOK8** | *H_o_* | 0.833 | 0.833 | 0.833 | 1.000 | 0.833 | 0.833 | 0.833 | 0.833 | 0.833 | 0.833 | 1.000 | 1.000 | 0.813 | 0.833 | 0.833 | 0.824 | 0.833 | 0.833 | 0.833 | 1.000 | 0.833 |
|  | *H_e_* | 0.486 | 0.486 | 0.486 | 0.500 | 0.486 | 0.486 | 0.486 | 0.486 | 0.486 | 0.486 | 0.500 | 0.500 | 0.482 | 0.486 | 0.486 | 0.484 | 0.486 | 0.486 | 0.486 | 0.500 | 0.486 |
|  | *F_is_* | **-0.714** | **-0.714** | **-0.714** | **-1.000** | **-0.714** | **-0.714** | **-0.714** | **-0.714** | **-0.714** | **-0.714** | **-1.000** | **-1.000** | **-0.684** | **-0.714** | **-0.714** | **-0.700** | **-0.714** | **-0.714** | **-0.714** | **-1.000** | **-0.714** |
| **btwnMtK** | *H_o_* | 1.000 | 1.000 | 1.000 | 1.000 | 1.000 | 1.000 | 1.000 | 1.000 | 1.000 | 1.000 | 1.000 | 1.000 | 1.000 | 1.000 | 1.000 | 1.000 | 1.000 | 1.000 | 1.000 | 1.000 | 1.000 |
|  | *H_e_* | 0.500 | 0.500 | 0.500 | 0.500 | 0.500 | 0.500 | 0.500 | 0.500 | 0.500 | 0.500 | 0.500 | 0.500 | 0.500 | 0.500 | 0.500 | 0.500 | 0.500 | 0.500 | 0.500 | 0.500 | 0.500 |
|  | *F_is_* | **-1.000** | **-1.000** | **-1.000** | **-1.000** | **-1.000** | **-1.000** | **-1.000** | **-1.000** | **-1.000** | **-1.000** | **-1.000** | **-1.000** | **-1.000** | **-1.000** | **-1.000** | **-1.000** | **-1.000** | **-1.000** | **-1.000** | **-1.000** | **-1.000** |

*H_o_*: observed heterozygosity; *H_e_*: expected heterozygosity; *F_is_*: inbreeding coefficient. Values in bold represent significant departures in Hardy-Weinberg Equilibrium. #N/A: not determined
