# Supplementary material for: Using host‐associated differentiation to track source population and dispersal distance among insect vectors of plant pathogens
Source: Evol Appl. 2019 Feb 12;12(4):692–704. doi: 10.1111/eva.12733 (PMC6439873; doi:10.1111/eva.12733)
Supplement: Supplementary file 8 [file EVA-12-692-s008.docx]

**Table S5.** Polymorphism and heterozygosity of year 1 (2012) and year 2 (2013) *Aphis craccivora* populations, as assessed by Hardy Weinberg Equilibrium: dataset with repeat MLGs removed, after assignment by best-estimates method.

| **Population** | Column1 | **Ac101026** | **Ac102292** | **Ac105372** | **Ac106241** | **Ac107071** | **Ac19923** | **Ac24056** | **Ac24766** | **Ac25986** | **Ac33020** | **Ac45540** | **Ac65895** | **Ac66996** | **Ac80798** | **Ac82746** | **Ac85822** | **Ac88299** | **Ac91605** | **Ac9471** | **Ac94989** | **Ac99882** |
| --- | --- | --- | --- | --- | --- | --- | --- | --- | --- | --- | --- | --- | --- | --- | --- | --- | --- | --- | --- | --- | --- | --- |
| **pan_2012** | *H_o_* | 0.200 | 0.200 | 0.200 | 0.500 | 0.200 | 0.200 | 0.600 | 0.200 | 0.200 | 0.250 | 0.400 | 0.800 | 0.250 | 0.200 | 0.400 | 0.200 | 0.400 | 0.600 | 0.200 | 0.200 | 0.200 |
|  | *H_e_* | 0.180 | 0.180 | 0.180 | 0.375 | 0.180 | 0.180 | 0.420 | 0.420 | 0.180 | 0.219 | 0.320 | 0.480 | 0.219 | 0.180 | 0.480 | 0.180 | 0.320 | 0.420 | 0.420 | 0.180 | 0.180 |
|  | *F_is_* | -0.111 | -0.111 | -0.111 | -0.333 | -0.111 | -0.111 | -0.429 | 0.524 | -0.111 | -0.143 | -0.250 | -0.667 | -0.143 | -0.111 | 0.167 | -0.111 | -0.250 | -0.429 | 0.524 | -0.111 | -0.111 |
| **WI-locust_2012** | *H_o_* | 0.500 | 0.500 | 0.000 | 0.000 | 0.000 | 0.000 | 0.500 | 0.000 | 0.000 | 0.000 | 0.000 | 0.000 | 0.000 | 0.000 | 0.000 | 0.000 | 0.000 | 0.500 | 0.000 | 0.500 | 0.000 |
|  | *H_e_* | 0.375 | 0.375 | 0.000 | 0.000 | 0.000 | 0.000 | 0.375 | 0.000 | 0.000 | 0.000 | 0.000 | 0.000 | 0.000 | 0.000 | 0.000 | 0.000 | 0.000 | 0.375 | 0.000 | 0.375 | 0.000 |
|  | *F_is_* | -0.333 | -0.333 | #N/A | #N/A | #N/A | #N/A | -0.333 | #N/A | #N/A | #N/A | #N/A | #N/A | #N/A | #N/A | #N/A | #N/A | #N/A | -0.333 | #N/A | -0.333 | #N/A |
| **KY-locust_2012** | *H_o_* | 0.333 | 0.333 | 0.000 | 0.333 | 0.000 | 0.000 | 0.333 | 0.000 | 0.000 | 0.000 | 0.000 | 0.000 | 0.000 | 0.000 | 0.000 | 0.000 | 0.000 | 0.333 | 0.000 | 0.333 | 0.000 |
|  | *H_e_* | 0.278 | 0.278 | 0.000 | 0.278 | 0.000 | 0.000 | 0.278 | 0.000 | 0.000 | 0.000 | 0.000 | 0.000 | 0.000 | 0.000 | 0.000 | 0.000 | 0.000 | 0.278 | 0.000 | 0.278 | 0.000 |
|  | *F_is_* | -0.200 | -0.200 | #N/A | -0.200 | #N/A | #N/A | -0.200 | #N/A | #N/A | #N/A | #N/A | #N/A | #N/A | #N/A | #N/A | #N/A | #N/A | -0.200 | #N/A | -0.200 | #N/A |
| **IL-locust_2012** | *H_o_* | 0.500 | 0.500 | 0.000 | 0.000 | 0.000 | 0.000 | 0.500 | 0.000 | 0.000 | 0.000 | 0.000 | 0.000 | 0.000 | 0.000 | 0.000 | 0.000 | 0.000 | 0.500 | 0.000 | 0.500 | 0.000 |
|  | *H_e_* | 0.375 | 0.375 | 0.000 | 0.000 | 0.000 | 0.000 | 0.375 | 0.000 | 0.000 | 0.000 | 0.000 | 0.000 | 0.000 | 0.000 | 0.000 | 0.000 | 0.000 | 0.375 | 0.000 | 0.375 | 0.000 |
|  | *F_is_* | -0.333 | -0.333 | #N/A | #N/A | #N/A | #N/A | -0.333 | #N/A | #N/A | #N/A | #N/A | #N/A | #N/A | #N/A | #N/A | #N/A | #N/A | -0.333 | #N/A | -0.333 | #N/A |
| **MI-locust_2012** | *H_o_* | 0.000 | 0.000 | 0.000 | 0.000 | 0.000 | 0.000 | 0.000 | 0.000 | 0.000 | 0.000 | 0.000 | 0.000 | 0.000 | 0.000 | 0.000 | 0.000 | 0.000 | 0.000 | 0.000 | 0.000 | 0.000 |
|  | *H_e_* | 0.000 | 0.000 | 0.000 | 0.000 | 0.000 | 0.000 | 0.000 | 0.000 | 0.000 | 0.000 | 0.000 | 0.000 | 0.000 | 0.000 | 0.000 | 0.000 | 0.000 | 0.000 | 0.000 | 0.000 | 0.000 |
|  | *F_is_* | #N/A | #N/A | #N/A | #N/A | #N/A | #N/A | #N/A | #N/A | #N/A | #N/A | #N/A | #N/A | #N/A | #N/A | #N/A | #N/A | #N/A | #N/A | #N/A | #N/A | #N/A |
| **625&231_2012** | *H_o_* | 0.000 | 0.000 | 0.000 | 0.000 | 0.000 | 0.000 | 0.000 | 0.000 | 0.000 | 0.000 | 0.000 | 0.000 | 0.000 | 0.000 | 0.000 | 0.000 | 0.000 | 0.000 | 0.000 | 0.000 | 0.000 |
|  | *H_e_* | 0.000 | 0.000 | 0.000 | 0.000 | 0.000 | 0.000 | 0.000 | 0.000 | 0.000 | 0.000 | 0.000 | 0.000 | 0.000 | 0.000 | 0.000 | 0.000 | 0.000 | 0.000 | 0.000 | 0.000 | 0.000 |
|  | *F_is_* | #N/A | #N/A | #N/A | #N/A | #N/A | #N/A | #N/A | #N/A | #N/A | #N/A | #N/A | #N/A | #N/A | #N/A | #N/A | #N/A | #N/A | #N/A | #N/A | #N/A | #N/A |
| **SR16&280W_2012** | *H_o_* | 0.333 | 0.333 | 0.000 | 0.333 | 0.000 | 0.000 | 0.333 | 0.000 | 0.000 | 0.000 | 0.000 | 0.000 | 0.000 | 0.000 | 0.000 | 0.000 | 0.000 | 0.333 | 0.000 | 0.333 | 0.000 |
|  | *H_e_* | 0.278 | 0.278 | 0.000 | 0.278 | 0.000 | 0.000 | 0.278 | 0.000 | 0.000 | 0.000 | 0.000 | 0.000 | 0.000 | 0.000 | 0.000 | 0.000 | 0.000 | 0.278 | 0.000 | 0.278 | 0.000 |
|  | *F_is_* | -0.200 | -0.200 | #N/A | -0.200 | #N/A | #N/A | -0.200 | #N/A | #N/A | #N/A | #N/A | #N/A | #N/A | #N/A | #N/A | #N/A | #N/A | -0.200 | #N/A | -0.200 | #N/A |
| **Hwy43&I-65_2012** | *H_o_* | 0.000 | 0.000 | 0.000 | 0.000 | 0.000 | 0.000 | 0.000 | 0.000 | 0.000 | 0.000 | 0.000 | 0.000 | 0.000 | 0.000 | 0.000 | 0.000 | 0.000 | 0.000 | 0.000 | 0.000 | 0.000 |
|  | *H_e_* | 0.000 | 0.000 | 0.000 | 0.000 | 0.000 | 0.000 | 0.000 | 0.000 | 0.000 | 0.000 | 0.000 | 0.000 | 0.000 | 0.000 | 0.000 | 0.000 | 0.000 | 0.000 | 0.000 | 0.000 | 0.000 |
|  | *F_is_* | #N/A | #N/A | #N/A | #N/A | #N/A | #N/A | #N/A | #N/A | #N/A | #N/A | #N/A | #N/A | #N/A | #N/A | #N/A | #N/A | #N/A | #N/A | #N/A | #N/A | #N/A |
| **Meridian&218E_2012** | *H_o_* | 0.000 | 0.000 | 0.000 | 0.000 | 0.000 | 0.000 | 0.000 | 0.000 | 0.000 | 0.000 | 0.000 | 0.000 | 0.000 | 0.000 | 0.000 | 0.000 | 0.000 | 0.000 | 0.000 | 0.000 | 0.000 |
|  | *H_e_* | 0.000 | 0.000 | 0.000 | 0.000 | 0.000 | 0.000 | 0.000 | 0.000 | 0.000 | 0.000 | 0.000 | 0.000 | 0.000 | 0.000 | 0.000 | 0.000 | 0.000 | 0.000 | 0.000 | 0.000 | 0.000 |
|  | *F_is_* | #N/A | #N/A | #N/A | #N/A | #N/A | #N/A | #N/A | #N/A | #N/A | #N/A | #N/A | #N/A | #N/A | #N/A | #N/A | #N/A | #N/A | #N/A | #N/A | #N/A | #N/A |
| **Hwy52&Walnut_2012** | *H_o_* | 0.500 | 0.500 | 0.500 | 0.500 | 0.500 | 0.500 | 0.500 | 0.500 | 0.500 | 0.500 | 0.500 | 0.500 | 0.500 | 0.500 | 0.500 | 0.500 | 0.500 | 0.500 | 0.500 | 0.500 | 0.500 |
|  | *H_e_* | 0.375 | 0.375 | 0.375 | 0.375 | 0.375 | 0.375 | 0.375 | 0.375 | 0.375 | 0.375 | 0.375 | 0.375 | 0.375 | 0.375 | 0.375 | 0.375 | 0.375 | 0.375 | 0.375 | 0.375 | 0.375 |
|  | *F_is_* | -0.333 | -0.333 | -0.333 | -0.333 | -0.333 | -0.333 | -0.333 | -0.333 | -0.333 | -0.333 | -0.333 | -0.333 | -0.333 | -0.333 | -0.333 | -0.333 | -0.333 | -0.333 | -0.333 | -0.333 | -0.333 |
| **IN-OH_2012** | *H_o_* | 0.000 | 0.000 | 0.000 | 0.000 | 0.000 | 0.000 | 0.000 | 0.000 | 0.000 | 0.000 | 0.000 | 0.000 | 0.000 | 0.000 | 0.000 | 0.000 | 0.000 | 0.000 | 0.000 | 0.000 | 0.000 |
|  | *H_e_* | 0.000 | 0.000 | 0.000 | 0.000 | 0.000 | 0.000 | 0.000 | 0.000 | 0.000 | 0.000 | 0.000 | 0.000 | 0.000 | 0.000 | 0.000 | 0.000 | 0.000 | 0.000 | 0.000 | 0.000 | 0.000 |
|  | *F_is_* | #N/A | #N/A | #N/A | #N/A | #N/A | #N/A | #N/A | #N/A | #N/A | #N/A | #N/A | #N/A | #N/A | #N/A | #N/A | #N/A | #N/A | #N/A | #N/A | #N/A | #N/A |
| **MI-clover_2012** | *H_o_* | 1.000 | 1.000 | 1.000 | 1.000 | 1.000 | 1.000 | 1.000 | 1.000 | 1.000 | 1.000 | 1.000 | 1.000 | 1.000 | 1.000 | 1.000 | 1.000 | 1.000 | 1.000 | 1.000 | 1.000 | 1.000 |
|  | *H_e_* | 0.500 | 0.500 | 0.500 | 0.500 | 0.500 | 0.500 | 0.500 | 0.500 | 0.500 | 0.500 | 0.500 | 0.500 | 0.500 | 0.500 | 0.500 | 0.500 | 0.500 | 0.500 | 0.500 | 0.500 | 0.500 |
|  | *F_is_* | -1.000 | -1.000 | -1.000 | -1.000 | -1.000 | -1.000 | -1.000 | -1.000 | -1.000 | -1.000 | -1.000 | -1.000 | -1.000 | -1.000 | -1.000 | -1.000 | -1.000 | -1.000 | -1.000 | -1.000 | -1.000 |
| **Chelsea_2012** | *H_o_* | 1.000 | 1.000 | 1.000 | 1.000 | 1.000 | 1.000 | 1.000 | 1.000 | 1.000 | 1.000 | 1.000 | 1.000 | 1.000 | 1.000 | 1.000 | 1.000 | 1.000 | 1.000 | 1.000 | 1.000 | 1.000 |
|  | *H_e_* | 0.500 | 0.500 | 0.500 | 0.500 | 0.500 | 0.500 | 0.500 | 0.500 | 0.500 | 0.500 | 0.500 | 0.500 | 0.500 | 0.500 | 0.500 | 0.500 | 0.500 | 0.500 | 0.500 | 0.500 | 0.500 |
|  | *F_is_* | -1.000 | -1.000 | -1.000 | -1.000 | -1.000 | -1.000 | -1.000 | -1.000 | -1.000 | -1.000 | -1.000 | -1.000 | -1.000 | -1.000 | -1.000 | -1.000 | -1.000 | -1.000 | -1.000 | -1.000 | -1.000 |
| **BuckCreek2-3_2012** | *H_o_* | 0.200 | 0.200 | 0.200 | 0.800 | 0.200 | 0.200 | 0.400 | 0.600 | 0.200 | 0.200 | 0.400 | 0.600 | 0.200 | 0.200 | 0.200 | 0.200 | 0.400 | 0.600 | 0.200 | 0.200 | 0.200 |
|  | *H_e_* | 0.180 | 0.180 | 0.180 | 0.480 | 0.180 | 0.180 | 0.320 | 0.500 | 0.180 | 0.180 | 0.480 | 0.420 | 0.180 | 0.180 | 0.500 | 0.180 | 0.320 | 0.420 | 0.500 | 0.180 | 0.180 |
|  | *F_is_* | -0.111 | -0.111 | -0.111 | -0.667 | -0.111 | -0.111 | -0.250 | -0.200 | -0.111 | -0.111 | 0.167 | -0.429 | -0.111 | -0.111 | 0.600 | -0.111 | -0.250 | -0.429 | 0.600 | -0.111 | -0.111 |
| **Otterbein_2012** | *H_o_* | 1.000 | 1.000 | 1.000 | 1.000 | 1.000 | 1.000 | 1.000 | 1.000 | 1.000 | 1.000 | 1.000 | 1.000 | 1.000 | 1.000 | 1.000 | 1.000 | 1.000 | 1.000 | 1.000 | 1.000 | 1.000 |
|  | *H_e_* | 0.500 | 0.500 | 0.500 | 0.500 | 0.500 | 0.500 | 0.500 | 0.500 | 0.500 | 0.500 | 0.500 | 0.500 | 0.500 | 0.500 | 0.500 | 0.500 | 0.500 | 0.500 | 0.500 | 0.500 | 0.500 |
|  | *F_is_* | -1.000 | -1.000 | -1.000 | -1.000 | -1.000 | -1.000 | -1.000 | -1.000 | -1.000 | -1.000 | -1.000 | -1.000 | -1.000 | -1.000 | -1.000 | -1.000 | -1.000 | -1.000 | -1.000 | -1.000 | -1.000 |
| **USDA-Madison_2012** | *H_o_* | 0.000 | 0.000 | 0.000 | 0.000 | 0.000 | 0.000 | 0.000 | 0.000 | 0.000 | 0.000 | 0.000 | 0.000 | 0.000 | 0.000 | 0.000 | 0.000 | 0.000 | 0.000 | 0.000 | 0.000 | 0.000 |
|  | *H_e_* | 0.000 | 0.000 | 0.000 | 0.000 | 0.000 | 0.000 | 0.000 | 0.000 | 0.000 | 0.000 | 0.000 | 0.000 | 0.000 | 0.000 | 0.000 | 0.000 | 0.000 | 0.000 | 0.000 | 0.000 | 0.000 |
|  | *F_is_* | #N/A | #N/A | #N/A | #N/A | #N/A | #N/A | #N/A | #N/A | #N/A | #N/A | #N/A | #N/A | #N/A | #N/A | #N/A | #N/A | #N/A | #N/A | #N/A | #N/A | #N/A |
| **WI_2012** | *H_o_* | 1.000 | 1.000 | 1.000 | 1.000 | 1.000 | 1.000 | 1.000 | 1.000 | 1.000 | 1.000 | 1.000 | 1.000 | 1.000 | 1.000 | 1.000 | 1.000 | 1.000 | 1.000 | 1.000 | 1.000 | 1.000 |
|  | *H_e_* | 0.500 | 0.500 | 0.500 | 0.500 | 0.500 | 0.500 | 0.500 | 0.500 | 0.500 | 0.500 | 0.500 | 0.500 | 0.500 | 0.500 | 0.500 | 0.500 | 0.500 | 0.500 | 0.500 | 0.500 | 0.500 |
|  | *F_is_* | -1.000 | -1.000 | -1.000 | -1.000 | -1.000 | -1.000 | -1.000 | -1.000 | -1.000 | -1.000 | -1.000 | -1.000 | -1.000 | -1.000 | -1.000 | -1.000 | -1.000 | -1.000 | -1.000 | -1.000 | -1.000 |
| **IL_2012** | *H_o_* | 1.000 | 1.000 | 1.000 | 1.000 | 1.000 | 1.000 | 1.000 | 1.000 | 1.000 | 1.000 | 1.000 | 1.000 | 1.000 | 1.000 | 1.000 | 1.000 | 1.000 | 1.000 | 1.000 | 1.000 | 1.000 |
|  | *H_e_* | 0.500 | 0.500 | 0.500 | 0.500 | 0.500 | 0.500 | 0.500 | 0.500 | 0.500 | 0.500 | 0.500 | 0.500 | 0.500 | 0.500 | 0.500 | 0.500 | 0.500 | 0.500 | 0.500 | 0.500 | 0.500 |
|  | *F_is_* | -1.000 | -1.000 | -1.000 | -1.000 | -1.000 | -1.000 | -1.000 | -1.000 | -1.000 | -1.000 | -1.000 | -1.000 | -1.000 | -1.000 | -1.000 | -1.000 | -1.000 | -1.000 | -1.000 | -1.000 | -1.000 |
| **KY_2012** | *H_o_* | 1.000 | 1.000 | 1.000 | 1.000 | 1.000 | 1.000 | 1.000 | 1.000 | 1.000 | 1.000 | 1.000 | 1.000 | 1.000 | 1.000 | 1.000 | 1.000 | 1.000 | 1.000 | 1.000 | 1.000 | 1.000 |
|  | *H_e_* | 0.500 | 0.500 | 0.500 | 0.500 | 0.500 | 0.500 | 0.500 | 0.500 | 0.500 | 0.500 | 0.500 | 0.500 | 0.500 | 0.500 | 0.500 | 0.500 | 0.500 | 0.500 | 0.500 | 0.500 | 0.500 |
|  | *F_is_* | -1.000 | -1.000 | -1.000 | -1.000 | -1.000 | -1.000 | -1.000 | -1.000 | -1.000 | -1.000 | -1.000 | -1.000 | -1.000 | -1.000 | -1.000 | -1.000 | -1.000 | -1.000 | -1.000 | -1.000 | -1.000 |
| **TPAC_2012** | *H_o_* | 1.000 | 1.000 | 1.000 | 1.000 | 1.000 | 1.000 | 1.000 | 1.000 | 1.000 | 1.000 | 1.000 | 1.000 | 1.000 | 1.000 | 1.000 | 1.000 | 1.000 | 1.000 | 1.000 | 1.000 | 1.000 |
|  | *H_e_* | 0.500 | 0.500 | 0.500 | 0.500 | 0.500 | 0.500 | 0.500 | 0.500 | 0.500 | 0.500 | 0.500 | 0.500 | 0.500 | 0.500 | 0.500 | 0.500 | 0.500 | 0.500 | 0.500 | 0.500 | 0.500 |
|  | *F_is_* | -1.000 | -1.000 | -1.000 | -1.000 | -1.000 | -1.000 | -1.000 | -1.000 | -1.000 | -1.000 | -1.000 | -1.000 | -1.000 | -1.000 | -1.000 | -1.000 | -1.000 | -1.000 | -1.000 | -1.000 | -1.000 |
| **WieseN_2012** | *H_o_* | 1.000 | 1.000 | 1.000 | 1.000 | 1.000 | 1.000 | 1.000 | 1.000 | 1.000 | 1.000 | 1.000 | 1.000 | 1.000 | 1.000 | 1.000 | 1.000 | 1.000 | 1.000 | 1.000 | 1.000 | 1.000 |
|  | *H_e_* | 0.500 | 0.500 | 0.500 | 0.500 | 0.500 | 0.500 | 0.500 | 0.500 | 0.500 | 0.500 | 0.500 | 0.500 | 0.500 | 0.500 | 0.500 | 0.500 | 0.500 | 0.500 | 0.500 | 0.500 | 0.500 |
|  | *F_is_* | -1.000 | -1.000 | -1.000 | -1.000 | -1.000 | -1.000 | -1.000 | -1.000 | -1.000 | -1.000 | -1.000 | -1.000 | -1.000 | -1.000 | -1.000 | -1.000 | -1.000 | -1.000 | -1.000 | -1.000 | -1.000 |
| **Cole7_2012** | *H_o_* | 1.000 | 1.000 | 1.000 | 1.000 | 1.000 | 1.000 | 1.000 | 1.000 | 1.000 | 1.000 | 1.000 | 1.000 | 1.000 | 1.000 | 1.000 | 1.000 | 1.000 | 1.000 | 1.000 | 1.000 | 1.000 |
|  | *H_e_* | 0.500 | 0.500 | 0.500 | 0.500 | 0.500 | 0.500 | 0.500 | 0.500 | 0.500 | 0.500 | 0.500 | 0.500 | 0.500 | 0.500 | 0.500 | 0.500 | 0.500 | 0.500 | 0.500 | 0.500 | 0.500 |
|  | *F_is_* | -1.000 | -1.000 | -1.000 | -1.000 | -1.000 | -1.000 | -1.000 | -1.000 | -1.000 | -1.000 | -1.000 | -1.000 | -1.000 | -1.000 | -1.000 | -1.000 | -1.000 | -1.000 | -1.000 | -1.000 | -1.000 |
| **Buck1_2012** | *H_o_* | 1.000 | 1.000 | 1.000 | 1.000 | 1.000 | 1.000 | 1.000 | 1.000 | 1.000 | 1.000 | 1.000 | 1.000 | 1.000 | 1.000 | 1.000 | 1.000 | 1.000 | 1.000 | 1.000 | 1.000 | 1.000 |
|  | *H_e_* | 0.500 | 0.500 | 0.500 | 0.500 | 0.500 | 0.500 | 0.500 | 0.500 | 0.500 | 0.500 | 0.500 | 0.500 | 0.500 | 0.500 | 0.500 | 0.500 | 0.500 | 0.500 | 0.500 | 0.500 | 0.500 |
|  | *F_is_* | -1.000 | -1.000 | -1.000 | -1.000 | -1.000 | -1.000 | -1.000 | -1.000 | -1.000 | -1.000 | -1.000 | -1.000 | -1.000 | -1.000 | -1.000 | -1.000 | -1.000 | -1.000 | -1.000 | -1.000 | -1.000 |
| **Crosby_2012** | *H_o_* | 1.000 | 1.000 | 1.000 | 1.000 | 1.000 | 1.000 | 1.000 | 1.000 | 1.000 | 1.000 | 1.000 | 1.000 | 1.000 | 1.000 | 1.000 | 1.000 | 1.000 | 1.000 | 1.000 | 1.000 | 1.000 |
|  | *H_e_* | 0.500 | 0.500 | 0.500 | 0.500 | 0.500 | 0.500 | 0.500 | 0.500 | 0.500 | 0.500 | 0.500 | 0.500 | 0.500 | 0.500 | 0.500 | 0.500 | 0.500 | 0.500 | 0.500 | 0.500 | 0.500 |
|  | *F_is_* | -1.000 | -1.000 | -1.000 | -1.000 | -1.000 | -1.000 | -1.000 | -1.000 | -1.000 | -1.000 | -1.000 | -1.000 | -1.000 | -1.000 | -1.000 | -1.000 | -1.000 | -1.000 | -1.000 | -1.000 | -1.000 |
| **MI_2012** | *H_o_* | 1.000 | 1.000 | 1.000 | 1.000 | 1.000 | 1.000 | 1.000 | 1.000 | 1.000 | 1.000 | 1.000 | 1.000 | 1.000 | 1.000 | 1.000 | 1.000 | 1.000 | 1.000 | 1.000 | 1.000 | 1.000 |
|  | *H_e_* | 0.500 | 0.500 | 0.500 | 0.500 | 0.500 | 0.500 | 0.500 | 0.500 | 0.500 | 0.500 | 0.500 | 0.500 | 0.500 | 0.500 | 0.500 | 0.500 | 0.500 | 0.500 | 0.500 | 0.500 | 0.500 |
|  | *F_is_* | -1.000 | -1.000 | -1.000 | -1.000 | -1.000 | -1.000 | -1.000 | -1.000 | -1.000 | -1.000 | -1.000 | -1.000 | -1.000 | -1.000 | -1.000 | -1.000 | -1.000 | -1.000 | -1.000 | -1.000 | -1.000 |
| **pan_2013** | *H_o_* | 0.087 | 0.087 | 0.043 | 0.619 | 0.087 | 0.043 | 0.261 | 0.364 | 0.043 | 0.043 | 0.391 | 0.571 | 0.043 | 0.043 | 0.409 | 0.048 | 0.087 | 0.565 | 0.435 | 0.130 | 0.043 |
|  | *H_e_* | 0.083 | 0.083 | 0.043 | 0.482 | 0.083 | 0.043 | 0.287 | 0.483 | 0.043 | 0.043 | 0.466 | 0.408 | 0.043 | 0.043 | 0.499 | 0.046 | 0.083 | 0.496 | 0.491 | 0.122 | 0.043 |
|  | *F_is_* | -0.045 | -0.045 | -0.022 | -0.285 | -0.045 | -0.022 | 0.092 | 0.248 | -0.022 | -0.022 | 0.160 | -0.400 | -0.022 | -0.022 | 0.180 | -0.024 | -0.045 | -0.139 | 0.115 | -0.070 | -0.022 |
| **MIbl** | *H_o_* | 0.000 | 0.000 | 0.000 | 0.500 | 0.000 | 0.000 | 0.000 | 0.000 | 0.000 | 0.000 | 0.000 | 0.000 | 0.000 | 0.000 | 0.000 | 0.000 | 0.000 | 0.000 | 0.000 | 0.000 | 0.000 |
|  | *H_e_* | 0.000 | 0.000 | 0.000 | 0.375 | 0.000 | 0.000 | 0.000 | 0.000 | 0.000 | 0.000 | 0.000 | 0.000 | 0.000 | 0.000 | 0.000 | 0.000 | 0.000 | 0.000 | 0.000 | 0.000 | 0.000 |
|  | *F_is_* | #N/A | #N/A | #N/A | -0.333 | #N/A | #N/A | #N/A | #N/A | #N/A | #N/A | #N/A | #N/A | #N/A | #N/A | #N/A | #N/A | #N/A | #N/A | #N/A | #N/A | #N/A |
| **OHbl** | *H_o_* | 0.200 | 0.400 | 0.200 | 0.600 | 0.600 | 0.200 | 0.600 | 0.400 | 0.200 | 0.200 | 0.600 | 0.400 | 0.200 | 0.200 | 0.800 | 0.200 | 0.200 | 0.400 | 0.600 | 0.200 | 0.200 |
|  | *H_e_* | 0.180 | 0.320 | 0.180 | 0.420 | 0.420 | 0.180 | 0.420 | 0.320 | 0.180 | 0.180 | 0.420 | 0.320 | 0.180 | 0.180 | 0.480 | 0.180 | 0.180 | 0.320 | 0.420 | 0.180 | 0.180 |
|  | *F_is_* | -0.111 | -0.250 | -0.111 | -0.429 | -0.429 | -0.111 | -0.429 | -0.250 | -0.111 | -0.111 | -0.429 | -0.250 | -0.111 | -0.111 | -0.667 | -0.111 | -0.111 | -0.250 | -0.429 | -0.111 | -0.111 |
| **KYbl** | *H_o_* | 0.000 | 0.000 | 0.000 | 0.500 | 0.000 | 0.000 | 0.000 | 0.000 | 0.000 | 0.000 | 0.000 | 0.000 | 0.000 | 0.000 | 0.000 | 0.000 | 0.000 | 0.000 | 0.000 | 0.000 | 0.000 |
|  | *H_e_* | 0.000 | 0.000 | 0.000 | 0.375 | 0.000 | 0.000 | 0.000 | 0.000 | 0.000 | 0.000 | 0.000 | 0.000 | 0.000 | 0.000 | 0.000 | 0.000 | 0.000 | 0.000 | 0.000 | 0.000 | 0.000 |
|  | *F_is_* | #N/A | #N/A | #N/A | -0.333 | #N/A | #N/A | #N/A | #N/A | #N/A | #N/A | #N/A | #N/A | #N/A | #N/A | #N/A | #N/A | #N/A | #N/A | #N/A | #N/A | #N/A |
| **wcINbl** | *H_o_* | 0.000 | 0.000 | 0.000 | 0.500 | 0.000 | 0.000 | 0.000 | 0.000 | 0.000 | 0.000 | 0.000 | 0.000 | 0.000 | 0.000 | 0.000 | 0.000 | 0.000 | 0.000 | 0.000 | 0.000 | 0.000 |
|  | *H_e_* | 0.000 | 0.000 | 0.000 | 0.375 | 0.000 | 0.000 | 0.000 | 0.000 | 0.000 | 0.000 | 0.000 | 0.000 | 0.000 | 0.000 | 0.000 | 0.000 | 0.000 | 0.000 | 0.000 | 0.000 | 0.000 |
|  | *F_is_* | #N/A | #N/A | #N/A | -0.333 | #N/A | #N/A | #N/A | #N/A | #N/A | #N/A | #N/A | #N/A | #N/A | #N/A | #N/A | #N/A | #N/A | #N/A | #N/A | #N/A | #N/A |
| **nearMTbl3** | *H_o_* | 0.000 | 0.000 | 0.000 | 0.000 | 0.000 | 0.000 | 0.000 | 0.000 | 0.000 | 0.000 | 0.000 | 0.000 | 0.000 | 0.000 | 0.000 | 0.000 | 0.000 | 0.000 | 0.000 | 0.000 | 0.000 |
|  | *H_e_* | 0.000 | 0.000 | 0.000 | 0.000 | 0.000 | 0.000 | 0.000 | 0.000 | 0.000 | 0.000 | 0.000 | 0.000 | 0.000 | 0.000 | 0.000 | 0.000 | 0.000 | 0.000 | 0.000 | 0.000 | 0.000 |
|  | *F_is_* | #N/A | #N/A | #N/A | #N/A | #N/A | #N/A | #N/A | #N/A | #N/A | #N/A | #N/A | #N/A | #N/A | #N/A | #N/A | #N/A | #N/A | #N/A | #N/A | #N/A | #N/A |
| **CarolCoIN** | *H_o_* | 0.000 | 0.000 | 0.000 | 0.000 | 0.000 | 0.000 | 0.000 | 0.000 | 0.000 | 0.000 | 0.000 | 0.000 | 0.000 | 0.000 | 0.000 | 0.000 | 0.000 | 0.000 | 0.000 | 0.000 | 0.000 |
|  | *H_e_* | 0.000 | 0.000 | 0.000 | 0.000 | 0.000 | 0.000 | 0.000 | 0.000 | 0.000 | 0.000 | 0.000 | 0.000 | 0.000 | 0.000 | 0.000 | 0.000 | 0.000 | 0.000 | 0.000 | 0.000 | 0.000 |
|  | *F_is_* | #N/A | #N/A | #N/A | #N/A | #N/A | #N/A | #N/A | #N/A | #N/A | #N/A | #N/A | #N/A | #N/A | #N/A | #N/A | #N/A | #N/A | #N/A | #N/A | #N/A | #N/A |
| **nearOKbl** | *H_o_* | 0.000 | 0.000 | 0.000 | 0.500 | 0.000 | 0.000 | 0.000 | 0.000 | 0.000 | 0.000 | 0.000 | 0.000 | 0.000 | 0.000 | 0.000 | 0.000 | 0.000 | 0.000 | 0.000 | 0.000 | 0.000 |
|  | *H_e_* | 0.000 | 0.000 | 0.000 | 0.375 | 0.000 | 0.000 | 0.000 | 0.000 | 0.000 | 0.000 | 0.000 | 0.000 | 0.000 | 0.000 | 0.000 | 0.000 | 0.000 | 0.000 | 0.000 | 0.000 | 0.000 |
|  | *F_is_* | #N/A | #N/A | #N/A | -0.333 | #N/A | #N/A | #N/A | #N/A | #N/A | #N/A | #N/A | #N/A | #N/A | #N/A | #N/A | #N/A | #N/A | #N/A | #N/A | #N/A | #N/A |
| **btwnMObl** | *H_o_* | 0.250 | 0.250 | 0.500 | 0.500 | 0.250 | 0.250 | 0.250 | 0.250 | 0.250 | 0.250 | 0.250 | 0.250 | 0.250 | 0.250 | 0.250 | 0.250 | 0.250 | 0.250 | 0.250 | 0.250 | 0.250 |
|  | *H_e_* | 0.219 | 0.219 | 0.375 | 0.375 | 0.219 | 0.219 | 0.219 | 0.219 | 0.219 | 0.219 | 0.219 | 0.219 | 0.219 | 0.219 | 0.219 | 0.219 | 0.219 | 0.219 | 0.219 | 0.219 | 0.219 |
|  | *F_is_* | -0.143 | -0.143 | -0.333 | -0.333 | -0.143 | -0.143 | -0.143 | -0.143 | -0.143 | -0.143 | -0.143 | -0.143 | -0.143 | -0.143 | -0.143 | -0.143 | -0.143 | -0.143 | -0.143 | -0.143 | -0.143 |
| **KnoxIL** | *H_o_* | 1.000 | 1.000 | 1.000 | 1.000 | 1.000 | 1.000 | 1.000 | 1.000 | 1.000 | 1.000 | 1.000 | 1.000 | 1.000 | 1.000 | 1.000 | 1.000 | 1.000 | 1.000 | 1.000 | 1.000 | 1.000 |
|  | *H_e_* | 0.500 | 0.500 | 0.500 | 0.500 | 0.500 | 0.500 | 0.500 | 0.500 | 0.500 | 0.500 | 0.500 | 0.500 | 0.500 | 0.500 | 0.500 | 0.500 | 0.500 | 0.500 | 0.500 | 0.500 | 0.500 |
|  | *F_is_* | -1.000 | -1.000 | -1.000 | -1.000 | -1.000 | -1.000 | -1.000 | -1.000 | -1.000 | -1.000 | -1.000 | -1.000 | -1.000 | -1.000 | -1.000 | -1.000 | -1.000 | -1.000 | -1.000 | -1.000 | -1.000 |
| **ArgyleWI** | *H_o_* | 0.500 | 0.500 | 0.500 | 0.500 | 0.500 | 0.500 | 1.000 | 1.000 | 0.500 | 0.500 | 0.500 | 1.000 | 0.500 | 0.500 | 1.000 | 0.500 | 1.000 | 0.500 | 1.000 | 0.500 | 0.500 |
|  | *H_e_* | 0.375 | 0.375 | 0.375 | 0.375 | 0.375 | 0.375 | 0.500 | 0.500 | 0.375 | 0.375 | 0.375 | 0.500 | 0.375 | 0.375 | 0.500 | 0.375 | 0.500 | 0.375 | 0.500 | 0.375 | 0.375 |
|  | *F_is_* | -0.333 | -0.333 | -0.333 | -0.333 | -0.333 | -0.333 | -1.000 | -1.000 | -0.333 | -0.333 | -0.333 | -1.000 | -0.333 | -0.333 | -1.000 | -0.333 | -1.000 | -0.333 | -1.000 | -0.333 | -0.333 |
| **PawPawMI** | *H_o_* | 1.000 | 1.000 | 1.000 | 1.000 | 1.000 | 1.000 | 1.000 | 1.000 | 1.000 | 1.000 | 1.000 | 1.000 | 1.000 | 1.000 | 1.000 | 1.000 | 1.000 | 1.000 | 1.000 | 1.000 | 1.000 |
|  | *H_e_* | 0.500 | 0.500 | 0.500 | 0.500 | 0.500 | 0.500 | 0.500 | 0.500 | 0.500 | 0.500 | 0.500 | 0.500 | 0.500 | 0.500 | 0.500 | 0.500 | 0.500 | 0.500 | 0.500 | 0.500 | 0.500 |
|  | *F_is_* | -1.000 | -1.000 | -1.000 | -1.000 | -1.000 | -1.000 | -1.000 | -1.000 | -1.000 | -1.000 | -1.000 | -1.000 | -1.000 | -1.000 | -1.000 | -1.000 | -1.000 | -1.000 | -1.000 | -1.000 | -1.000 |
| **sIN** | *H_o_* | 0.500 | 0.500 | 0.500 | 0.500 | 0.500 | 0.500 | 0.500 | 0.500 | 0.500 | 1.000 | 1.000 | 1.000 | 0.500 | 0.500 | 0.500 | 0.500 | 0.500 | 0.500 | 0.500 | 0.500 | 0.500 |
|  | *H_e_* | 0.375 | 0.375 | 0.375 | 0.375 | 0.375 | 0.375 | 0.375 | 0.375 | 0.375 | 0.500 | 0.500 | 0.500 | 0.375 | 0.375 | 0.375 | 0.375 | 0.375 | 0.375 | 0.375 | 0.375 | 0.375 |
|  | *F_is_* | -0.333 | -0.333 | -0.333 | -0.333 | -0.333 | -0.333 | -0.333 | -0.333 | -0.333 | -1.000 | -1.000 | -1.000 | -0.333 | -0.333 | -0.333 | -0.333 | -0.333 | -0.333 | -0.333 | -0.333 | -0.333 |
| **wcIN** | *H_o_* | 0.500 | 0.500 | 0.500 | 1.000 | 0.500 | 0.500 | 0.500 | 0.500 | 0.500 | 0.500 | 0.500 | 0.500 | 0.500 | 0.500 | 0.500 | 0.500 | 0.500 | 0.500 | 0.500 | 0.500 | 0.500 |
|  | *H_e_* | 0.375 | 0.375 | 0.375 | 0.500 | 0.375 | 0.375 | 0.375 | 0.375 | 0.375 | 0.375 | 0.375 | 0.375 | 0.375 | 0.375 | 0.375 | 0.375 | 0.375 | 0.375 | 0.375 | 0.375 | 0.375 |
|  | *F_is_* | -0.333 | -0.333 | -0.333 | -1.000 | -0.333 | -0.333 | -0.333 | -0.333 | -0.333 | -0.333 | -0.333 | -0.333 | -0.333 | -0.333 | -0.333 | -0.333 | -0.333 | -0.333 | -0.333 | -0.333 | -0.333 |
| **nearMT** | *H_o_* | 1.000 | 1.000 | 1.000 | 1.000 | 1.000 | 1.000 | 1.000 | 1.000 | 1.000 | 1.000 | 1.000 | 1.000 | 1.000 | 1.000 | 1.000 | 1.000 | 1.000 | 1.000 | 1.000 | 1.000 | 1.000 |
|  | *H_e_* | 0.500 | 0.500 | 0.500 | 0.500 | 0.500 | 0.500 | 0.500 | 0.500 | 0.500 | 0.500 | 0.500 | 0.500 | 0.500 | 0.500 | 0.500 | 0.500 | 0.500 | 0.500 | 0.500 | 0.500 | 0.500 |
|  | *F_is_* | -1.000 | -1.000 | -1.000 | -1.000 | -1.000 | -1.000 | -1.000 | -1.000 | -1.000 | -1.000 | -1.000 | -1.000 | -1.000 | -1.000 | -1.000 | -1.000 | -1.000 | -1.000 | -1.000 | -1.000 | -1.000 |
| **nearM** | *H_o_* | 0.667 | 0.667 | 0.333 | 0.667 | 0.333 | 0.333 | 0.667 | 0.333 | 0.333 | 0.333 | 0.667 | 0.667 | 0.333 | 0.333 | 0.667 | 0.333 | 0.333 | 1.000 | 0.333 | 0.667 | 0.333 |
|  | *H_e_* | 0.444 | 0.444 | 0.278 | 0.444 | 0.278 | 0.278 | 0.444 | 0.500 | 0.278 | 0.278 | 0.444 | 0.444 | 0.278 | 0.278 | 0.444 | 0.278 | 0.278 | 0.500 | 0.500 | 0.444 | 0.278 |
|  | *F_is_* | -0.500 | -0.500 | -0.200 | -0.500 | -0.200 | -0.200 | -0.500 | 0.333 | -0.200 | -0.200 | -0.500 | -0.500 | -0.200 | -0.200 | -0.500 | -0.200 | -0.200 | -1.000 | 0.333 | -0.500 | -0.200 |
| **nearOK10** | *H_o_* | 1.000 | 1.000 | 1.000 | 1.000 | 1.000 | 1.000 | 1.000 | 1.000 | 1.000 | 1.000 | 1.000 | 1.000 | 1.000 | 1.000 | 1.000 | 1.000 | 1.000 | 1.000 | 1.000 | 1.000 | 1.000 |
|  | *H_e_* | 0.500 | 0.500 | 0.500 | 0.500 | 0.500 | 0.500 | 0.500 | 0.500 | 0.500 | 0.500 | 0.500 | 0.500 | 0.500 | 0.500 | 0.500 | 0.500 | 0.500 | 0.500 | 0.500 | 0.500 | 0.500 |
|  | *F_is_* | -1.000 | -1.000 | -1.000 | -1.000 | -1.000 | -1.000 | -1.000 | -1.000 | -1.000 | -1.000 | -1.000 | -1.000 | -1.000 | -1.000 | -1.000 | -1.000 | -1.000 | -1.000 | -1.000 | -1.000 | -1.000 |
| **nearOK8** | *H_o_* | 0.500 | 0.500 | 0.500 | 1.000 | 0.500 | 0.500 | 0.500 | 0.500 | 0.500 | 0.500 | 1.000 | 1.000 | 0.500 | 0.500 | 0.500 | 0.500 | 0.500 | 0.500 | 0.500 | 1.000 | 0.500 |
|  | *H_e_* | 0.375 | 0.375 | 0.375 | 0.500 | 0.375 | 0.375 | 0.375 | 0.375 | 0.375 | 0.375 | 0.500 | 0.500 | 0.375 | 0.375 | 0.375 | 0.375 | 0.375 | 0.375 | 0.375 | 0.500 | 0.375 |
|  | *F_is_* | -0.333 | -0.333 | -0.333 | -1.000 | -0.333 | -0.333 | -0.333 | -0.333 | -0.333 | -0.333 | -1.000 | -1.000 | -0.333 | -0.333 | -0.333 | -0.333 | -0.333 | -0.333 | -0.333 | -1.000 | -0.333 |
| **btwnMtK** | *H_o_* | 1.000 | 1.000 | 1.000 | 1.000 | 1.000 | 1.000 | 1.000 | 1.000 | 1.000 | 1.000 | 1.000 | 1.000 | 1.000 | 1.000 | 1.000 | 1.000 | 1.000 | 1.000 | 1.000 | 1.000 | 1.000 |
|  | *H_e_* | 0.500 | 0.500 | 0.500 | 0.500 | 0.500 | 0.500 | 0.500 | 0.500 | 0.500 | 0.500 | 0.500 | 0.500 | 0.500 | 0.500 | 0.500 | 0.500 | 0.500 | 0.500 | 0.500 | 0.500 | 0.500 |
|  | *F_is_* | -1.000 | -1.000 | -1.000 | -1.000 | -1.000 | -1.000 | -1.000 | -1.000 | -1.000 | -1.000 | -1.000 | -1.000 | -1.000 | -1.000 | -1.000 | -1.000 | -1.000 | -1.000 | -1.000 | -1.000 | -1.000 |
|  |  |  |  |  |  |  |  |  |  |  |  |  |  |  |  |  |  |  |  |  |  |  |

*H_o_*: observed heterozygosity; *H_e_*: expected heterozygosity; *F_is_*: inbreeding coefficient. Values in bold represent significant departures in Hardy-Weinberg Equilibrium. #N/A: not determined
