# Supplementary material for: Using host‐associated differentiation to track source population and dispersal distance among insect vectors of plant pathogens
Source: Evol Appl. 2019 Feb 12;12(4):692–704. doi: 10.1111/eva.12733 (PMC6439873; doi:10.1111/eva.12733)
Supplement: Supplementary file 9 [file EVA-12-692-s009.docx]

**Table S6.** Multilocus disequilibrium estimates $\bar{r}$*_d_* for 999 randomized datasets of year 1 (2012) and year 2 (2013) combined, generated from generated from a full dataset of all *Aphis craccivora* populations, or population by host-association (black locust, alfalfa, clover). Datasets tested with repeat MLGs (a), or excluding effects of clonality with all repeat MLGs removed after assignment by a best-estimates (b) or conservative (c) method.

|  |  | $\bar{r}$*_d_* |  |
| --- | --- | --- | --- |
| Full dataset | 0.81_a_ ^**^ | 0.14_b_^**^ | 0.50_c_^**^ |
| Alfalfa populations | 0.81_a_ ^**^ | 0.18_b_^**^ | 0.54_c_^**^ |
| Black locust populations | 0.67_a_ ^**^ | 0.21_b_^**^ | 0.74_c_^**^ |
| Clover populations | 0.83_a_ ^**^ | 0.19_b_^**^ | 0.38_c_^**^ |

^**^indicates statistical significance (*P*<0.01) after 999 permutations of *Φ_PT_*, an analogue of *F_ST_*
